# Motor Vehicle Registration Taxes (MVRT) across EU countries: MNEs’ profitability and the role of market concentration

**DOI:** 10.1007/s10657-023-09775-8

**Published:** 2023-07-19

**Authors:** Pranvera Shehaj, Martin Zagler

**Affiliations:** 1 WU Vienna University of Economics and Business, Vienna, Austria; 2 UPO University of Eastern Piedmont, Novara, Italy

**Keywords:** Motor Vehicle Registration Tax (MVRT), Market concentration, Tax incidence, Profitability, Multinational enterprises, EU motor vehicle industry, H22, L11, L12, L13, L62

## Abstract

This paper discusses the effects of one-off Motor Vehicle Registration Taxes (MVRT) and market concentration level on the profitability of multinational enterprises (MNEs) operating in the European Union motor vehicle industry. Our simple theoretical framework shows that firm profits depend on the demand function and therefore on taxes applied to prices. We overcome empirically the challenges of making informative theoretical predictions on the pass-through rate under imperfect competition. We find that MVRT,—both as ad valorem taxes and as specific taxes,—have a significant negative effect on MNEs’ profitability. Our findings show a statistically significant positive effect of market concentration on profitability. Finally, our results suggest that the degree of competitiveness in the motor vehicle market moderates the effect of MVRT on firm profitability only in EU countries where the MVRT is an ad valorem tax, with the negative effect of the ad valorem MVRT becoming higher as the motor vehicle market becomes less competitive.

## Introduction

Arguably, a motor vehicle is the most significant household purchase of a tradable good. Therefore, the motor vehicle market could be a highly visible indicator of European market integration and is as such the focus of intense scrutiny (Dvir and Strasser, 2018, p.4). For this reason, and despite exempting the passenger motor vehicle market from the unrestricted competition article of the EU treaty, the European Commission (EC) has taken concrete steps to integrate the national markets and reduce price dispersion *(ibidem)*.^1^ However, literature provides strong evidence of cross-country price differentiation, which mostly grounds on the heterogeneity of consumer preferences and regulation within the EU (Dvir and Strasser, 2018; OECD, 2020). Differences in local costs and differences in local mark-ups explain why even in the context of an integrated EU market, car firms charge different prices across markets. To the extent that local costs differ across national markets, firms have incentives to charge different prices for otherwise identical models. A first source of local cost differences are the large and persistent car tax differences across countries (Goldberg and Verboven, 2004), i.e., differences in value added tax (VAT) and in additional taxes, such as Motor Vehicle Registration Taxes (MVRT),^2^ special car taxes, and environmental taxes.

In the EU market, there has been no harmonization or even approximation of taxes or tax rates on motor vehicles. Vehicle taxes differ greatly among countries by the amount charged, the method by which they are calculated, and the policy measures context for which the vehicle taxes are used (see Kunert and Kuhfeld, 2007; Ryan et al., 2009; Ajanovic and Haas, 2017; Hooftman et al., 2018; Wappelhorst et al., 2018; etc.). While an EU-wide vehicle tax does not exist, most EU countries impose a range of taxes on motor vehicles, which include: (i) a vehicle registration tax and value added tax (VAT); (ii) an annual, road tax or circulation tax; (iii) excise duties on fuel (diesel and petrol). The initial duty payable in all European Union countries on the purchase and first-time registration of a new motor vehicle^3^ is the value added tax (VAT), which remains the largest source of government revenue.^4^ Except of VAT, the other duties payable on the first-time registration of a new motor vehicle, are moderate fees and a registration tax (OECD, 2020, pp.170–171). Different factors serve as a basis for registration taxes in Europe (Kalinowska et al., 2009). They vary considerably from one country to the other, and member states tax motor vehicles mainly on a combination of different factors^5^ (ACEA Tax guide, 2020).

This paper focuses on the one-off Motor Vehicle Registration Taxes related to the first registration of motor vehicles across European Union member states. OECD (2020) considers motor vehicle registration as an exemplar of how motor vehicle taxation can affect the functioning of the motor vehicle market, as well as how large differences in tax system reinforce motor vehicle market fragmentation. In contrast with taxes on most other goods, motor vehicle taxes are to be paid in the country where the motor vehicle is registered. Given that motor vehicles need to register with a unique identification number in the principal country of use, the international differences in taxation of sales and registration of motor vehicles do not give rise to considerable cross-border shopping. Motor vehicles marketed in one country with specifications designed to meet the national tax structure^6^ are imperfect substitutes and may not effectively compete with motor vehicles sold in another country with different tax requirements (OECD, 2020, pp. 170–171).

On these premises, this study investigates whether Motor Vehicle Registration Taxes (MVRT) affect the market performance of multinational enterprises (MNEs) in the Sale of Motor Vehicles’ industry across EU countries. In addition, we analyze whether the impact of Motor Vehicle Registration Taxes on motor vehicle MNEs’ profitability depends on the concentration level in the national motor vehicle market where the customers purchase and register their motor vehicle. We investigate both questions while taking into account the heterogeneity in the formulation of Motor Vehicle Registration Taxes across EU countries, i.e., as an ad valorem tax on the net or gross price, or as a specific tax.

There is a large body of literature, more extensively reviewed in the next section, which indicates that imperfect competition may lead to consumer’s surplus being either higher or lower than under perfect competition (Katz and Rosen, 1985; Seade, 1980, 1985; etc.,). Similarly, empirical work, which has looked at the indirect tax^7^ burden on consumer prices in several industries, suggests mixed results (Carbonnier, 2007; Kosonen, 2015; etc.). However, empirical studies on tax incidence have provided limited evidence on the effects of indirect taxes on outcomes other than prices. Notable exceptions are Kosonen (2015), which suggests that a large VAT cut on hairdressers in Finland, increases hairdressers’ profits significantly; and Benzarti and Carloni (2019), which suggests that a large VAT cut for French sit-down restaurants tends to benefit firm owners with limited “trickle down” to consumers or employees. Overall, prior literature leaves open, first, whether the inconclusiveness about the role of imperfect competition on price effects of indirect taxes extend from the within-country-industry analysis to a cross-country setting. Second, it leaves open whether this inconclusiveness reflects on outcomes other than prices, such as firm profitability, which is the next step in the chain to be affected by price-increases of price-decreases effects of taxes.

In this study, instead of focusing on motor vehicle prices, we look at the tax incidence on firm profitability. Second, we conduct a cross-country analysis and exploit both the difference in Motor Vehicle Registration Tax’ rates and structure across EU countries, as well as differences in national motor vehicle markets’ concentration levels. Third, to the best of our knowledge, ours is the first study, which investigates the motor vehicle industry with a particular focus on the incidence of taxes related to the registration of motor vehicles on firm profitability. We focus on Motor Vehicle Registration Taxes (MVRT) charged across seventeen European Union member states and look at whether increases in such taxes would affect foreign-owned subsidiaries’ profitability, as an implication of their motor vehicle price increases or decreases effect. We extend then the analysis by questioning whether the potential effect is heterogeneous across national motor vehicle markets’ concentration levels and whether motor vehicle multinationals are differently affected by registration taxes, depending on the market power that they possess in the country where the customers register their motor vehicle. Considering that the Motor Vehicle Registration Tax is formulated as an ad valorem tax on net or gross price in some EU countries and as a specific tax in others, we investigate whether the role of imperfect competition on the way in which motor vehicle MNEs respond to Motor Vehicle Registration Tax increases, differs across different formulations of the tax.

We build a simple theoretical framework showing that firm’s profits depend on the own price elasticity of demand, elasticity of the cost function, and total costs, which all depend on the demand function and therefore on taxes applied to prices. Since under imperfect competition the pass-through is not only determined by the elasticity of the supply and demand, but the curvature of the demand function also plays a role, and because standard demand forms restrict curvature of the demand function in ways that have little empirical or theoretical foundation imperfect competition makes it particularly difficult to credibly predict the pass-through rate. For the purposes of our study, we can say that it is the challenges of making informative theoretical predictions, which motivate empirical analysis of the incidence of one-off taxes related to the purchase and registration of motor vehicles, as well as their effect on firms’ profitability in a cross-country setting with focus on multinational enterprises.

In line with prior empirical studies, our results show that as the concentration level in the Sale of Motor Vehicles industry increases, this causes firms to exercise market power, becoming more efficient and generating larger profits. We find a statistically significant negative effect of Motor Vehicle Registration Taxes (MVRT) on the profitability of foreign-owned subsidiaries operating in the Sale of Motor Vehicle’ industry, both in EU countries where the MVRT is an ad valorem tax and in countries where it is a specific tax. The negative effect of MVRT on firm profitability may reflect a price-decrease effect of these taxes. As registration taxes become higher, firms tend to offer lower pre-tax prices in order to compensate for the higher tax effect. Our findings suggest that the role of imperfect competition as a moderator on the effect of the Motor Vehicle Registration Tax on firms’ market performance, depends on the structure of the tax, i.e., whether it is an ad valorem tax or a specific tax. We find that market concentration plays a role on the effect of MVRT on firm profitability only in countries where the MVRT is an ad valorem tax. The negative effect of an increase in the ad valorem MVRT becomes higher as the market becomes less competitive. Consequently, increases in the concentration level increase the tax burden borne by motor vehicle sellers, which reflects on lower profitability levels.

The remainder of the study is as follows. Section 2 provides a literature review of previous studies focused on the sales taxes shifting. Section 3 presents our theoretical framework. Section 4 explains the construction of our market concentration and firm profitability’s measures. Section 5 presents our data. Section 6 discloses the empirical strategy and results. Section 7 presents the robustness tests analysis, while Sect. 8 concludes.

## Literature review

### Imperfect competition and tax incidence

Several theoretical papers have studied the question of sales tax shifting on prices for a wide range of imperfect competition models (e.g., Cournot oligopoly model with conjectural variations, Bertrand oligopoly model with differentiated goods, etc.,), confirming that imperfect competition may lead to consumer’s share being either higher or lower than under perfect competition [Katz and Rosen (1985), Stern (1987) and Besley (1989), Seade (1980) Anderson et al*.,* (2001b)]. Seade (1985) suggested that, following a rise in excise taxation, and assuming linear costs, consumer’s price will accordingly rise to a greater extent than the shift in marginal cost, representing a more than 100% shift of the excise tax to consumers, if and only if the elasticity of the slope of inverse demand is greater than one. Stern (1987) shows that the price increasing effect of a tax will be higher in monopolistic competition compared to oligopolies, if and only if, taxes reduce profits for a given number of firms. Delipalla and Keen (1992) found that the consumer share of the tax burden is higher in the case of specific sales taxes, extending the predominantly view of ad valorem taxation as implying a low consumer price, relatively high tax revenue and low profits when the entry is precluded, from the monopoly case to the context of imperfect competition. Their results are amplified in Anderson et al*.,* (2001b). Authors suggest that in an oligopolistic industry with differentiated products and price setting (Bertrand firms), both taxes may be passed on to consumers by more than 100%, and an increase in the tax rate can increase short run firm profits, consequently, the long run number of firms.

Besley and Rosen (1999)^8^ was the first empirical study to test the tax shifting through a number of local sales tax variations in United States. Authors found that for a few goods, whose tax shifting on prices was found significantly different from 100%, commodity taxes are over-shifted, where a ten-cent increase in the revenue extracted from the sale of these commodities, led to an increase in their prices of more than a dime. Delipalla and O’ Donnell (2001), Bonnet and Réquillart (2013) and Carbonnier (2013) focus on testing empirically the economic theory statement that in an imperfectly competitive market, changes in per unit consumption taxes should induce a larger increase in prices than do ad valorem taxes. Respectively, these papers provide evidence of under-shifting of both taxes in the European cigarette industry, over-shifting of both taxes in the French soft drinks’ market, and over-shifting of specific taxes but under-shifting of ad valorem taxes, in the French alcoholic beverage market.

Other empirical papers, which focus on sales tax burden, although relatively few, provide results, which differ according to the industry under investigation. Investigating the alcoholic beverage market in Alaska,—known as a low-competition industry,—Kenkel (2005) provides evidence that alcoholic taxes are more than fully passed through to beverage prices. Carbonnier (2007) provides evidence of VAT shifting in two different markets in France: the new motor vehicle market, which was close to oligopoly, and the housing repair market, close to perfect competition. This paper suggests that consumers pay 77% of the VAT on housing repair services, while they pay a lower share of VAT on new motor vehicles, only 57%. Doyle and Samphantharak (2008) find less-than-full shifting of the gasoline sales tax in Illinois and Indiana. Alm et al., (2009), using monthly gasoline price data for U.S. states over the period 1984–1999, find strong and consistent evidence of full shifting of gasoline taxes to the final consumer. In addition, authors suggest that tax shifting depends in part on the degree of competition in a state, with less than full shifting in more rural less competitive U.S. states. Fuest et al., (2015) suggest that the share of the tax shifted to consumers in the Austrian gasoline market, increases significantly with the market power of the suppliers, and the tendency to shift taxes to consumers is significantly stronger in less competitive markets. Gaarder provides evidence from the high-concentrated food industry in Norway, suggesting that VAT on food items is completely shifted to consumer prices, implying that producers bear none of the tax burden.

### Market concentration and firm profitability

Literature unfolds two scenarios suggesting that firm profitability levels should be positively correlated with industry concentration levels. If markets are contestable, that is, few barriers to entry, then even firms operating in a country where this industry is highly concentrated should behave as if they have many competitors (Baumol, 1982). Consequently, profitability should not be affected by changes in industry concentration levels because the threat of potential entrants would keep markets competitive.^9^ More recently, Autor et al., (2017) propounds a model in which a higher degree of competition helps the most productive “super star” firms capture market share, thus increasing industry concentration. In overall, this strand of literature posits that intense quality competition may increase the total costs of operating in a particular industry, which in turn will lead to concentrated markets, as low price–cost margins reduce the number of market participants (Grullon et al., 2019, p. 707).

Alternatively, significant barriers to entry, including economies of scale, technological barriers, and large capital requirements,—as is the case of the automobile industry,—should cause firms operating in increasingly concentrated industry to exercise market power, hence becoming more efficient and generating larger abnormal profits (e.g., Bain, 1951). Barriers to entry in the form of government regulations, for example, could increase the profitability and market value of incumbent firms (Bessen, 2016). Firms operating in the motor vehicle industry have to deal with immense entry and exit barriers, where the existence of economies of scale is probably the most significant entry barrier. On the other hand, exit barriers, such as the heavy sunk costs, make it difficult for firms to enter the motor vehicle market. In addition, technological barriers, and large capital requirements challenge firm entry to the motor vehicle market.

### Contributions of the paper to the existing literature

Our work aims to modestly contribute to several gaps consulted both in the literature on indirect taxes’ incidence, as well as on the literature on motor vehicle taxation in general.

(i) First, to the best of our knowledge, empirical studies on tax incidence have provided limited evidence on the effects of sales taxes on changes on outcomes other than prices. Notable exceptions are Kosonen (2015), which suggests that a large VAT cut on hairdressers in Finland increases hairdressers’ profits significantly; and Benzarti and Carloni (2019), which suggests that a large cut VAT for French sit-down restaurants tend to benefit firm owners with limited “trickle down” to consumers or employees. Instead of focusing on motor vehicle prices, we look at the tax incidence on firm profitability, which is the next step in the chain to be affected by price-increases or price-decreases effects of taxes.
(ii) Second, cross-country empirical evidence on tax incidence is all but missing. An exception is Delipalla and O’ Donnell (2001), which studies the European cigarette industry, and Alm et al., (2009), which studies the U.S. gasoline market. However, both studies limit the analysis to the incidence of sales taxes on consumer prices. Instead, we conduct a cross-country analysis and exploit both the difference in Motor Vehicle Registration Tax’ rates and structure across EU countries, as well as differences in national motor vehicle markets’ concentration levels.
(iii) Third, the motor vehicle industry has never been under investigation with a focus on tax incidence, and in particular on the incidence of taxes related to the purchase and registration of motor vehicles on firm market performance. An exception is Carbonnier (2007), which however focuses on the impact of the value added tax on new motor vehicle prices. The stream of the literature on motor vehicle taxation has been mainly focused on the role of national fiscal policies, i.e., of different types of taxes (including vehicle registration tax), on the reduction of CO_2_ emissions from road transport, and on the de-carbonization of newly sold passenger motor vehicles (e.g. Alberini and Bareit, 2019; Cerruti et al., 2019; Gerlagh et al., 2018; Klier and Linn, 2015; Rogan et al., 2011; Ryan et al., 2009); while Ciccone (2018), Yan et al*.,* (2018) and Ciccone and Soldani (2019) focus on the impact of vehicle registration tax reforms in Norway on CO_2_ intensity and on new vehicles’ sales. We focus on Motor Vehicle Registration Taxes charged across seventeen European Union member states and look at whether increases in such taxes would affect foreign-owned subsidiaries’ profitability as an implication of their motor vehicle price increases or decreases effect. In addition, we question whether the potential effect is heterogeneous across national motor vehicle markets’ concentration levels and whether motor vehicle multinationals are differently affected by registration taxes, depending on the market power they possess in the country where the customers register their motor vehicle.

## Theory

Considering the consumer (or after-tax) price is p*(1 + t), where *p* is the producer price and *t* is the tax, and supposing that there are *n* firms and that each firm produces a variant of a differentiated product, firm *i*’s profit is given by:

1 $${\varvec{\uppi}}_{\mathbf{i}} = \frac{{{\text{p}}_{{\text{i}}} }}{{\left( {1 + {\text{t}}_{{\text{i}}} { }} \right)}}{\text{q}}_{{\text{i}}} - c\left( {{\text{q}}_{{\text{i}}} } \right)$$

where c (·) is the cost function common for each firm, q_i_ = D_i_(p_i_; p_-i_) is the demand for firm *i*’s product as a function of firm *i*’s own consumer price, p_i_ and a vector consisting of the other firms’ consumer prices (p_-i_). This function is continuously differentiable, decreasing in p_i_ and increasing in all elements of p_-i_. From (2) we calculate the change in firm *i*’s profits given a change in price p_i_ such as the FOC is satisfied:

2 $$\begin{aligned} \frac{{{\text{d}}\uppi_{{\text{i}}} }}{{{\text{dp}}_{{\text{i}}} }} = \frac{1}{{\left( {1 + {\text{t}}_{{\text{i}}} } \right)}}{\text{q}}_{{\text{i}}} + \frac{{{\text{p}}_{{\text{i}}} }}{{\left( {1 + {\text{t}}_{{\text{i}}} } \right)}}\frac{{{\text{dq}}_{{\text{i}}} }}{{{\text{dp}}_{{\text{i}}} }} - \frac{{{\text{dc}}_{{\text{i}}} }}{{{\text{dq}}_{{\text{i}}} }}\frac{{{\text{dq}}_{{\text{i}}} }}{{{\text{dp}}_{{\text{i}}} }}\, & = 0 \\ \left[ {{\text{p}}_{{\text{i}}} - \left( {1 + {\text{t}}_{{\text{i}}} } \right){ }\frac{{{\text{dc}}_{{\text{i}}} }}{{{\text{dq}}_{{\text{i}}} }}} \right]\frac{{{\text{dq}}_{{\text{i}}} }}{{{\text{dp}}_{{\text{i}}} }} + {\text{q}}_{{\text{i}}} & = 0 \\ \left[ {{1} - \left( {1 + {\text{t}}_{{\text{i}}} } \right)\frac{{{\text{dc}}_{{\text{i}}} }}{{{\text{dq}}_{{\text{i}}} }} \frac{1}{{{\text{p}}_{{\text{i}}} { }}}} \right]\frac{{{\text{dq}}_{{\text{i}}} }}{{{\text{dp}}_{{\text{i}}} }}\frac{{{\text{p}}_{{\text{i}}} }}{{{\text{q}}_{{\text{i}}} }} + 1 & = 0, \\ \end{aligned}$$

where $\frac{{\mathrm{dq}}_{\mathrm{i}}}{{\mathrm{dp}}_{\mathrm{i}} }\frac{{\mathrm{p}}_{\mathrm{i}}}{{\mathrm{q}}_{\mathrm{i}} }=$−ε > 0 is the own price elasticity of demand.

3 $$\begin{aligned} \left[ {1 - \left( {{\text{1 + t}}_{{\text{i}}} } \right)\frac{{{\text{dc}}_{{\text{i}}} }}{{{\text{dq}}_{{\text{i}}} }} \frac{1}{{{\text{p}}_{{\text{i}}} }}} \right]\upvarepsilon & = {1} \\ \left( {{\text{1 + t}}_{{\text{i}}} } \right)\frac{{{\text{dc}}_{{\text{i}}} }}{{{\text{dq}}_{{\text{i}}} }} \frac{1}{{{\text{p}}_{{\text{i}}} }} & = 1 - {1 \mathord{\left/ {\vphantom {1 \upvarepsilon }} \right. \kern-0pt} \upvarepsilon } \\ {\text{p}}_{{\text{i}}} { } & = \left( {{\text{1 + t}}_{{\text{i}}} } \right)\frac{\upvarepsilon }{\upvarepsilon - 1}\frac{{{\text{dc}}_{{\text{i}}} }}{{ {\text{dq}}_{{\text{i}}} }}, \\ \end{aligned}$$

which gives the Amoroso-Robinson rule as the price p_i_ as a mark-up depending on the own price elasticity, over marginal costs. Note that the own price elasticity of demand depends on prices set by competitors p_-i_, hence fiercer competition will reduce the mark-up. Substituting the Amoroso-Robinson rule (3) into the definition of profits (1) yields

4 $$\begin{aligned} {\varvec{\uppi}}_{\mathbf{i}} & = \frac{\upvarepsilon }{\upvarepsilon - 1}\frac{{{\text{dc}}_{{\text{i}}} }}{{{\text{dq}}_{{\text{i}}} { }}} {\text{q}}_{{\text{i}}} { } - {\text{c}}\left( {{\text{q}}_{{\text{i}}} { }} \right) \\ {\varvec{\uppi}}_{\mathbf{i}} & = \left[ {\frac{\upvarepsilon }{\upvarepsilon - 1}\frac{{{\text{dc}}_{{\text{i}}} }}{{{\text{dq}}_{{\text{i}}} { }}}\frac{{{\text{q}}_{{\text{i}}} }}{{{\text{c}}_{{\text{i}}} { }}} - 1} \right]{\text{c}}\left( {{\text{q}}_{{\text{i}}} { }} \right) \\ {\varvec{\uppi}}_{\mathbf{i}} & = \left[ {\frac{\upvarepsilon \upeta }{{\upvarepsilon - 1}} - 1} \right]{\text{c}}\left( {{\text{q}}_{{\text{i}}} { }} \right) \\ \end{aligned}$$

where η is the elasticity of the cost function. ε, η, and c all depend on the demand function D and therefore on taxes applied to prices.

Since under imperfect competition, the pass-through is not only determined by the elasticity of the supply and demand, but the curvature of the demand function also plays a role, and because standard demand forms restrict curvature of the demand function in ways that have little empirical or theoretical foundation (Weyl and Fabinger, 2013), imperfect competition makes it particularly difficult to credibly predict the pass-through rate. For the purposes of our study, we can say that it is the challenges of making informative theoretical predictions, which motivate empirical analysis of the incidence of one-off taxes related to the purchase and registration of motor vehicles, as well as their effect on firms’ profitability in a cross-country setting with focus on multinational enterprises.

In appendix C, we extend the model by specifying the demand function D. Under the assumption of constant elasticity of substitution (CES) and constant marginal costs, we show that firm i’s profits depend on ε, η, and c, as well as on the tax *t* on firm i’s motor vehicles and on the average tax *T* on competitors’ motor vehicles.

## Measuring market concentration and market performance

### Profitability measure for firm market performance

Three profitability measures are commonly used as measures of market performance^10^: Economic Profits or Rates of Return on Investment; Lerner Index or the Price–Cost Margin (PCM); and Tobin’s Q (see Aghion et al., 2005; Giroud and Mueller, 2010; Grullon et al., 2019; Gutierrez and Philippon, 2017; 2018; Sorbe and Johansson, 2017).^11^

For the purpose of our analysis, following Aghion et al., (2005), Sorbe and Johansson (2017) and Grullon et al., (2019), we choose the Lerner Index as a profitability measure, to proxy for motor vehicle foreign subsidiaries’ market performance across EU countries. This indicator is also known as the price–cost, and is defined as the distance between a firm’s price and marginal cost, i.e., (P-MC)/P. When prices exceed marginal cost, the Lerner index becomes positive and varies between zero and unity (Martins et al., 1996). It is closely related to the mark-up ratio, which measures the gap between the price and the marginal cost, determined as the markup for firm *f* in year* t*: μ_f,t_ = P_f,t_/MC_f,t_. Lerner is defined as follows: PCM_f,t_ = 1 − 1/μ_f,t_.

One obvious difficulty in computing the firm-level PCM is the impossibility to retrieve marginal cost measure from balance sheet data. To overcome these shortcomings, literature suggests two different methodologies for the calculation of the empirical Lerner Index at firm-level. With the first method, adopted by Aghion et al., (2005), following Nickell (1996), the operating revenue (net of depreciation and financial cost of capital) is divided by sales. With the second approach, developed by Tybout (2001), PCM is computed as sales net the expenditure on material and labor over sales, proxying marginal costs with variable cost.^12^ Given the data coverage and the purpose of our analysis, we follow the approach suggested in Aghion et al., (Tybout 2001), and stay in line with Gutierrez and Philippon (2018) and Grullon et al., (2019). In the empirical analysis, we measure Lerner Index as operating profits net of depreciation, provisions and an estimated financial cost of capital divided by *sales.* Given that in Orbis database *turnover* is more widely available compared to *sales*, and it is closely related to sales at the same time, in line with Sorbe and Johansson (2017), we calculate the Lerner index as operating profits *(item EBIT)* divided by operating revenue *(item Turnover)*. However, as a robustness check we scale the operating profits by firm’s sales as well.

### Measuring market concentration

Market concentration, also often referred to as industry concentration, refers to the extent to which the market shares of the largest firms within a market (industry) accounts for a large proportion of economic activity such as sales, assets, or employment.^13^ Discussions regarding the best measure of concentration usually conclude that the selection of a measure depends on the use to be made of the concentration estimate, and the nature of the data on which the estimate is based (Bailey and Boyle, 1971, p. 702). All concentration measures aim to capture the weight of the largest firms within an industry, but they differ in several aspects: in terms of what is “an industry”, in their definition of the “largest” and third, in their choice of denominator measuring activity of the whole industry (Bajgar et al*.,* 2019). In the following, we discuss our choice of concentration measure with respect to each of these aspects.

The economic analysis of concentration in this paper uses an *industry definition* based on the industry classification provided by the International Standard Industrial Classification of all Economic Activities (ISIC),^14^ fourth revision. We choose a high degree of disaggregation, a 3-digit industry level,^15^ i.e., *Section G, Division 45, Group 451: Sale of Motor Vehicles*.^16^ The underlying assumption is that firms sell one good and serve one industry defined at 3-digit in ISIC, fourth revision.

Market concentration measures typically fall into two general classes, i.e., discrete (e.g., CR1, CR4, CR8, etc.,) and cumulative indexes (e.g., Herfindahl (HF) and Hall—Tideman (HT) indexes). The principal type in the first class shows an absolute number of the largest firms in terms of sales (or their definition relies on the percentage of total industry output accounted for by the *n—*largest firms in a market). Cumulative measures instead, notably the Herfindahl–Hirschman index, which is a well-grounded measure in industrial organization theory (Tirole, 1988), take the number of firms in the industry into account, as well as the entire size distribution of the firms, and not just the share accounted for by *n—*largest number of firms. Although differences between measures of the two classes exist, they tend to yield reasonably comparable results (Bailey and Boyle, 1971, pp.702–703).

However, given that *concentration ratio (CR)* considers exclusively the relevance of the top—*n* firms and disregards the distribution of market shares of a given industry, it does not distinguish between markets in which, for instance, there are only four firms and those where there is a long tail of firms with smaller market shares. The *HH Index* solves this problem by calculating the square of the market share of each firm in the market and summing the resulting numbers, hence considering not only the equality of market shares across firms but also the number of firms in an industry (Cavalleri et al., 2019). A higher HHI implies weaker competition. In line with SCP studies,^17^ and following Kosonen (2015), Cavalleri et al., (2019), Grullon et al., (2019) and Bajgar et al*.,* (2019), in the main regressions, we use the *Herhindahl-Hirschman index*^18^ as our measure for the concentration level in the Sale of Motor Vehicles’ industry.^19^ Alternatively, as a robustness check we measure concentration as the share of industry sales due to the four largest firms (based on *sales*) in the industry, nominated CR4.^20^

The *industry denominator*’s choice for the construction of the market concentration measure has a striking effect on measured industry concentration trends. To construct market shares of firms in the defined industry, their sales should be scaled by the total sales in that industry.^21^ We use the total active firms’ sales operating in the Sale of Motor Vehicles’ industry as reported in Orbis database, for the seventeen EU countries, during the period 2011–2019. To construct the Herfindahl–Hirschman index using Orbis data within the three-digit country-industry-year, we sum up the squared ratios of firm sales to the total industry sales. We allow the index to vary between 0 and 10.000, taking the sum of the squared market shares as a percentage. If there is only one firm in the market, the HHI will equal 10.000; if the market is divided equally between a large number of firms, the HHI will approach zero. Herfindahl–Hirschman Index below 0.01 (or 100) indicates a highly competitive industry; below 0.15 (or 1500) indicates an un-concentrated industry; between 0.15 and 0.25 (or 1500 to 2500) indicates moderate concentration; above 0.25 (above 2500) indicates high concentration. However, sometimes less restrictive thresholds are used. According to the *Guidelines on the assessment of horizontal mergers under the Council Regulations on the control of concentrations between undertakings* (European Commission, 2002), the industry is *unconcentrated*, if the value of HHI is less than 0.1; *moderately concentrated*, if the value of HHI is in range [0.1; 0.2]; *highly concentrated*, if the value of HHI is greater than 0.2.

**Fig. 1:**
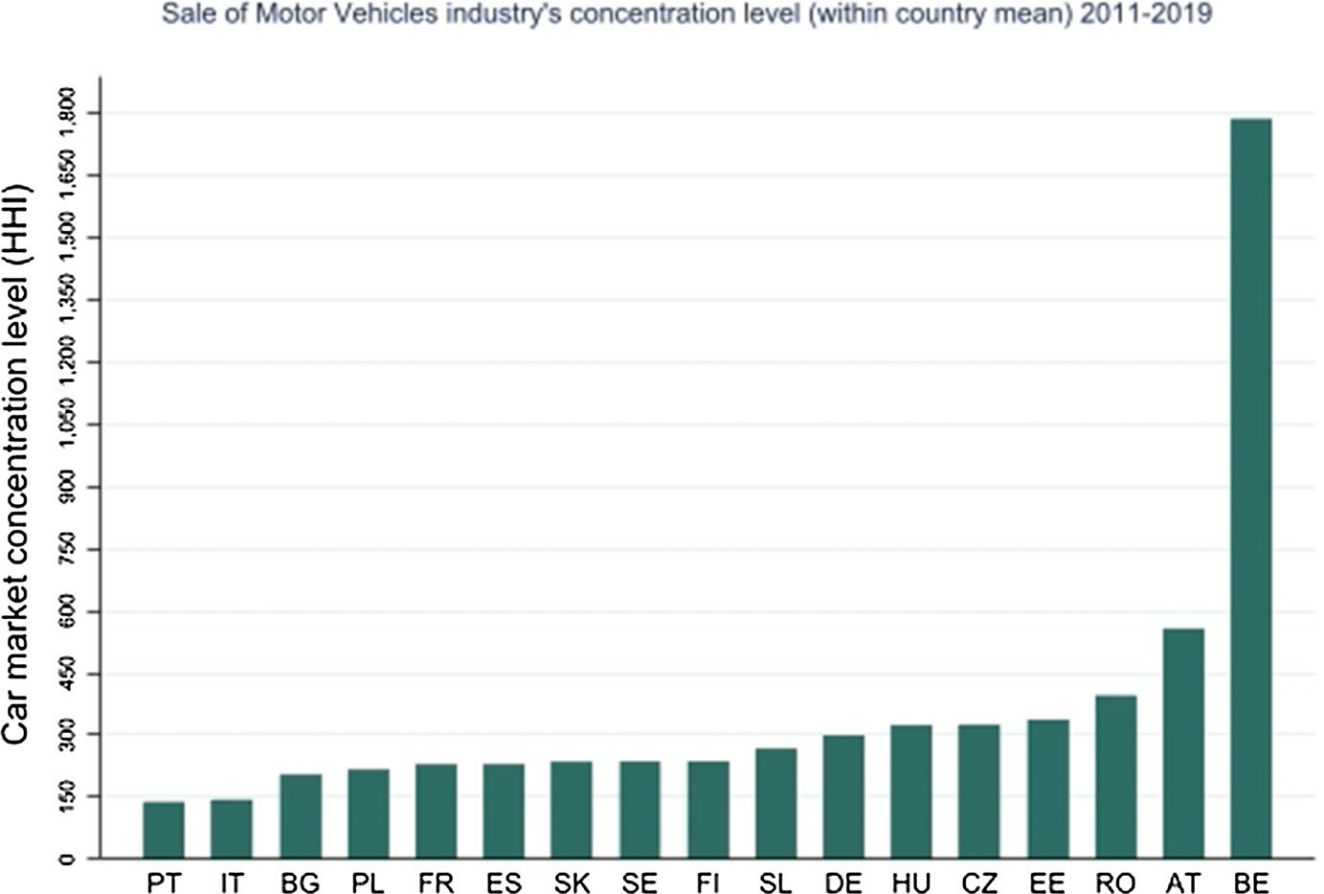
Within country mean of the Herfindahl–Hirschman Index in the Sale of Motor Vehicles’ industry, 2011–2019

Figure 1 shows the mean of the concentration level in the Sale of Motor Vehicles’ industry across countries, for the period 2011–2019, measured by the Herfindahl–Hirschman index. Countries are ranked in ascending order, from the most competitive, i.e., Portugal, where the mean of Herfindahl–Hirschman index takes the lowest value, of about 139.32, to the most concentrated, i.e., Belgium, where it takes the highest value, of about 1786.07. Belgium is the only country in our sample whose Herfindahl–Hirschman index in the motor vehicle industry indicates a moderate concentration. Its minimum value is 1636 points in 2015, while the highest value is observed in 2019, of about 1835. For the rest of the countries, the mean of HH index during the nine—year period is below 1500. In our dataset, during the period under analysis, concentration level in the motor vehicle market exhibits more variability in Austria and Belgium, respectively, between 437–774 and 1759–1834. Bulgaria, Romania, Sweden, and Portugal experience less variability.^22^

## Data

### Sample selection

The empirical analysis in this study is conducted using unconsolidated firm-level data of motor vehicle MNEs’ foreign-owned subsidiaries located in seventeen European Union member states. The data are obtained from the Bureau van Dijk’s Orbis database, which provides financial accounting statements for national and multinational companies worldwide. The observation period is from 2011 to 2019. The observation units of the analysis are the foreign-owned subsidiaries in the Sale of Motor Vehicles’ industry. We follow Schwellnus and Arnold (2008) and Marques and Pinho (2016), in detecting firms operating in the Sale of motor vehicles’ industry, which form part of multinational enterprises. A firm enters the sample if there is a multinational located in another country that owns at least 50% of the subsidiary’s capital. It enters the sample if it reports at least one subsidiary with a different value of the ‘subsidiary–country iso code’ variable than its own ‘country iso code’. In addition, a firm enters the sample if it reports a different value of the “global ultimate owner^23^–country iso code” variable.

The estimation sample is restricted to the incorporated firms. Practically, in Orbis this can be achieved by considering only firms with a strictly positive difference between reporting year and year of incorporation. Given that the earliest reporting year in our sample is 2011, we restrict the sample only to those firms with year of incorporation up to 2010, excluding firms with year of incorporation either later than 2010 or unknown (Schwellnus & Arnold, 2008). Many countries included in Orbis report exclusively data for firms with more than 20 employees or only a limited sample for firms under this threshold. To overcome concerns over the representativeness of the data set at the country level over time, which arise in this case, only firms displaying on average at least 20 employees over the period, were considered in the analysis (Calligaris et al*.,* 2018).^24^

### Definition of variables and summary statistics

Our dependent variable measuring foreign-owned subsidiaries’ market performance in the Sale of Motor Vehicles’ industry, is a profitability measure, i.e., the Lerner index, calculated as operating profits net of depreciation *(EBIT)* scaled by operating revenue *(Turnover)*.

The first variable of interest in our analysis is the one-off Motor Vehicle Registration Tax *(MVRT).* We use European Automobile Manufacturers’ Association (ACEA) Tax Guides (2011–2019) and OECD (2020) to look at the various criteria on which each country relies for the determination of the one-off registration taxes on motor vehicles. The significant heterogeneity across countries on the criteria used for the determination of the Motor Vehicle Registration Tax’ base and tax rates, as well as missing data reports on pre—and after-tax motor vehicle prices, led us towards an alternative approach for the calculation of motor vehicle registration duty per country and year. First, we relied on the European Automobile Manufacturers’ Association (ACEA) *Pocket Guides* (2011–2019) for data on new motor vehicle registrations^25^ by country and year. Secondly, we obtained data on Motor Vehicle Registration Tax revenues at general government level, reported in millions of euros, from the Eurostat Main National Accounts Tax Aggregates. We scaled the Motor Vehicle Registration Tax revenues by the number of new motor vehicle registrations, reaching an average of Motor Vehicle Registration Tax (in units of Euro), which customers pay per country and year.^26^

**Fig. 2:**
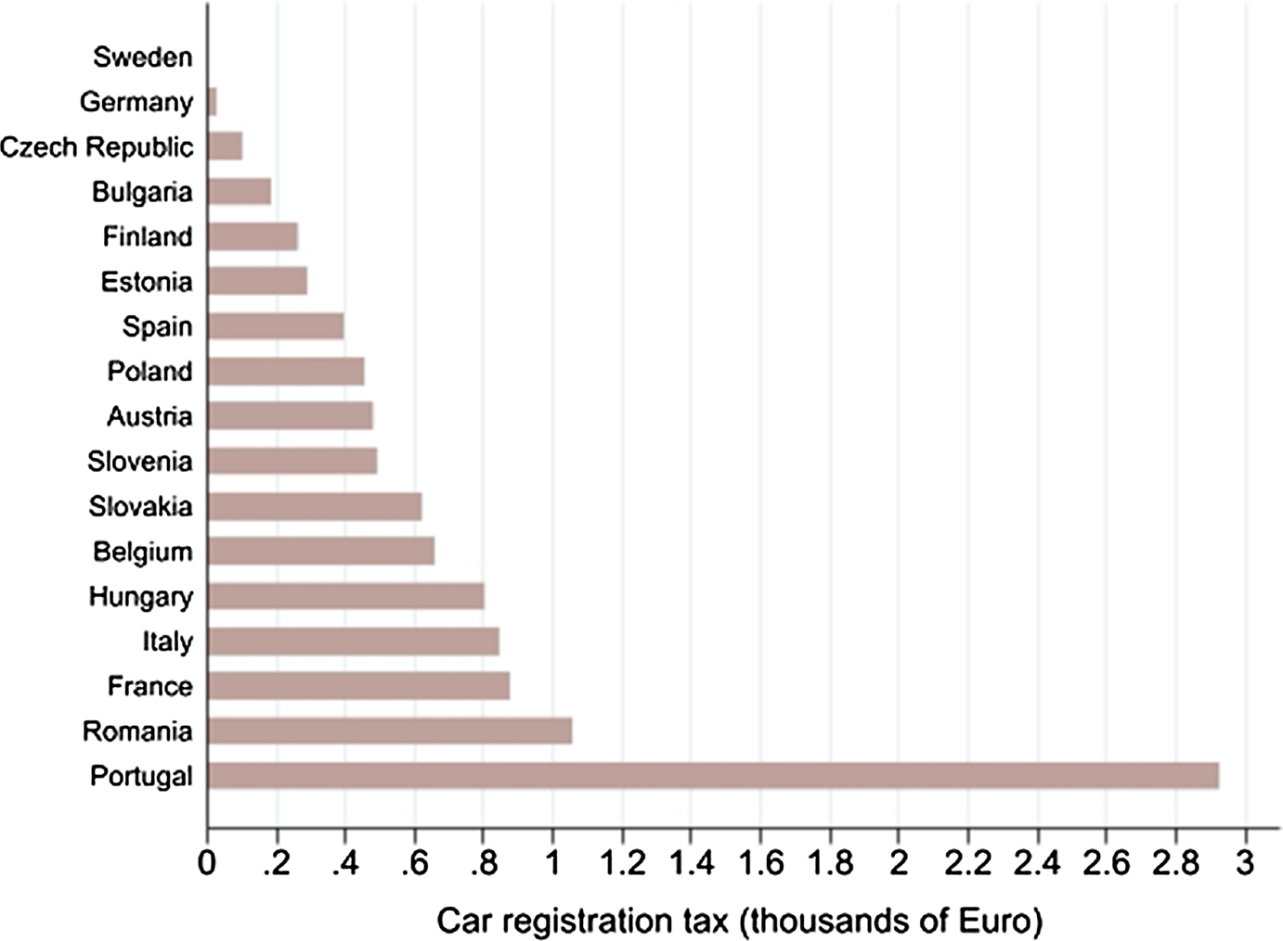
Within country mean of the Motor Vehicle Registration Tax (MVRT) on motor vehicles, 2011–2019

Figure 2 shows the distribution of the within country mean of the average Motor Vehicle Registration Tax in thousands of Euros for the period 2011–2019. Countries are ranked in ascending order from Sweden, where no registration tax is charged, to Portugal, which in our sample has the highest mean of Motor Vehicle Registration Tax across the nine-year period under analysis, of about 660.69 Euro.

Out of seventeen EU countries in our sample, Motor Vehicle Registration Tax is formulated as a *specific* tax in thirteen of them, and as an *ad valorem* tax levied either on the motor vehicle’s net or gross price in four of them, i.e., in Austria, Finland, Slovenia and Spain. For the purpose of our analysis, taking into consideration this distinction is relevant for theoretical and empirical reasons. Standard economic theory of tax incidence suggests that, when firms are competitive, specific taxation and ad valorem taxation are entirely equivalent. The meaning of equivalence is that a specific tax and an ad valorem tax leading to the same consumer price will raise the same amount of tax revenue. With imperfect competition this equivalence between the two forms of taxation breaks down (Hindrick and Myles, 2013, pp. 227–229). Empirical evidence has already tested the economic theory statement that in an imperfectly competitive market changes in per unit consumption taxes should induce a larger increase in prices than do ad valorem taxes (e.g., Delipalla and O’ Donnell, 2001; Bonnet and Réquillart, 2013; Carbonnier, 2013). Therefore, in our empirical estimations we distinguish between countries where the Motor Vehicle Registration Tax is formulated as an *ad valorem* tax and countries where it is formulated as a *specific* tax, in order to understand whether the structure of the MVRT across EU countries determines the impact of the MVRT on firm profitability, as well as the role of imperfect competition on the way firms responds to MVRT changes.

Our proxy variable for the concentration level in the Sale of Motor Vehicles’ industry in each country is the Herfindahl–Hirschman Index, while as a robustness check we also use the concentration ratio of the top—4 firms, based on sales, namely CR4.^27^

Additionally, in our empirical regression we control for the impact of the value added tax (VAT) on foreign-owned subsidiaries’ profitability in the EU Sale of Motor Vehicles’ industry. Being VAT the initial duty payable in all EU countries on the purchase of a new motor vehicle, is relevant for it to be included in an analysis of the impact of MVRT on firm profitability. We use the OECD Tax database and OECD (2020) to collect data on value added tax rates for the seventeen countries over the period 2011–2019.^28^ From the data, we observe that VAT remains constant across the nine-year period in nine out of seventeen countries in our sample, i.e., in Austria, Belgium, Bulgaria, Estonia, Germany, Poland, Portugal, Slovakia, Sweden. For the rest of the countries, small changes (mostly only once) occur between 2011 and 2019. Among the seventeen EU countries in our sample, the mean of VAT rate within the period 2011–2019 varies between 19% in Germany and 27% in Hungary.

As an additional control variable at country level, we include the statutory corporate income tax rate as well. Data for the statutory CIT per country and year are obtained from the OECD Tax database as well as KPMG. We use two firm-level control variables in the baseline regressions, i.e., firm-size, measured as the natural logarithm of total assets, and firm-age, measured as natural logarithm of the difference between current year and the year of incorporation.

Table 2 in Appendix A shows summary statistics of the constructed variables, as well as of their underlying variables. For the data cleaning process, we follow the framework proposed in Adams et al., (2019). First, we drop observations for which data on all the variables included in the baseline regressions is missing. Second, extreme values of the dependent and of independent variables, which may reflect reporting errors are identified and excluded from the sample.^29^ The selection criteria described in Sect. 5.1., as well as the data cleaning process, leaves us with 623 firms and 4995 firm-country-year observations. However, since the Lerner Index is a ratio, it can take on extreme values (in either direction) if the scaling variable becomes too small.^30^ Following Giroud and Mueller (2010), to mitigate the effect of outliers, we drop 1% of the firm-country-year observations at each tail of the Lerner index distribution.

## Estimations and main results

### Estimating the impact of the Motor Vehicle Registration Tax (MVRT) on firm profitability

First, we ask whether one-off Motor Vehicle Registration Taxes charged on the first registration of motor vehicles, have an impact on motor vehicle multinationals’ profitability. Second, we investigate whether the impact of Motor Vehicle Registration Taxes on motor vehicle sellers’ profitability depends on the concentration level in the motor vehicle market across EU countries. Consequently, whether increases in the Motor Vehicle Registration Tax affect the same MNE differently, depending on the concentration level of the motor vehicle industry in the national market where the motor vehicle is registered.

We exploit our panel-data to investigate each of the questions estimating an empirical model, which looks like the following:

5 $$\begin{aligned} {\text{Profitability}}_{{{\text{f}},{\text{c}},{\text{t}}}} & = \upbeta_{0} + \upbeta_{{1}} {\text{HHI}}_{{{\text{c}},{\text{t}}}} + \upbeta_{{2}} {\text{MVRT}}_{{{\text{c}},{\text{t}}}} + \updelta_{{1}} {\text{MVRT}}_{{{\text{c}},{\text{t}}}} *{\text{HHI}}_{{{\text{c}},{\text{t}}}} + \upbeta_{{3}} {\text{MVRT}}\_{\text{specific}}_{{{\text{c}},{\text{t}}}} \\ & \quad + \updelta_{{2}} {\text{MVRT}}_{{{\text{c}},{\text{t}}}} *{\text{MVRT}}\_{\text{specific}}_{{{\text{c}},{\text{t}}}} + \updelta_{{3}} {\text{MVRT}}_{{{\text{c}},{\text{t}}}} *{\text{MVRT}}\_{\text{specific}}_{{{\text{c}},{\text{t}}}} \\ & \quad *{\text{HHI}}_{{{\text{c}},{\text{t}} }} + \upbeta_{{4}} {\text{VAT}}_{{{\text{c}},{\text{t}}}} + \upbeta_{{5}} {\text{CIT}}_{{{\text{c}},{\text{t}} }} + \upbeta_{{6}} {\text{X}}_{{{\text{f}},{\text{c}},{\text{t}}}} + \upvarphi_{{\text{f}}} + \uptheta_{{\text{t}}} + \upvarepsilon_{{{\text{f}},{\text{c}},{\text{t}}}} \\ \end{aligned}$$

where *f* indexes firms, *c* indexes countries and *t* indexes time (year). Our left-hand variable, *Profitability*_*f,c,t,*_ is the net profit margin (or price–cost margin), i.e., the Lerner index, our main measure of the market performance of motor vehicle MNE’s subsidiary *f* operating in country *c* in year *t*. In our sample, the Lerner index varies between -9.7% and 18.1%. *MVRT*_*c,t*_, states for Motor Vehicle Registration Tax, calculated as the ratio of Motor Vehicle Registration Tax revenues and number of new motor vehicle registrations per country and year, and expressed in units of Euro.

*HHI*_*c,t*_ is our measure of concentration level in the motor vehicle industry in country *c* in year *t*. Based on the two scenarios suggested in literature discussed in Sect. 2.2, as well as on the characteristics that our industry under analysis exhibits, we expect for the effect of the industry concentration level on our dependent variable to be positive.

The interaction term *MVRT*_c,t_*HHI_*c,t*_ tests for an interaction between the effect of Motor Vehicle Registration Tax related to the first-time registration of motor vehicles on firms’ profitability and the motor vehicle market concentration level across EU countries.^31^ Thus, we want to know whether as the market becomes less competitive, and thus the concentration level in the Sale of Motor Vehicles’ industry increases, the effect of Motor Vehicle Registration Taxes on foreign-owned subsidiaries’ profitability changes. A statistically significant coefficient on the interaction term would suggest first, imperfect competition alters the impact of a tax increase on motor vehicle multinationals’ profitability. Consequently, the same motor vehicle MNE’s profits would be affected by a tax increase differently, depending on the market power that it possesses in the national motor vehicle market where the motor vehicle is registered. Second, it would suggest that price changes’ effects of a Motor Vehicle Registration Tax depend on the degree of the motor vehicle market competitiveness, which implies that European Union customers would be in an unequal position, where they bear different shares of the tax burden depending on the concentration level in the national market where they register their motor vehicle.

We introduce a dummy variable *MVRT_specific*_*c,t*_, in order to distinguish between countries where the Motor Vehicle Registration Tax is a *specific tax* or an *ad valorem tax* levied either on the net or gross price of motor vehicle. This dummy takes the value one in the former case and zero otherwise. We interact the *MVRT_specific*_*c,t*_ dummy with the Motor Vehicle Registration Tax variable, in order to look at the main effect of the *MVRT*_*c,t*_ as a specific tax on firm profitability. Second, we interact both *MVRT*_*c,t*_ and *MVRT_specific*_*c,t*_ with the market concentration measure, in order to investigate how market concentration alters the impact of Motor Vehicle Registration Tax on firms’ profitability when *MVRT* is a specific tax *vs* when it is formulated as an ad valorem tax.

We control for the impact of the value added tax (VAT_c,t_) on motor vehicle multinationals’ market performance, as measured by their profitability. CIT_c,t_ controls for the statutory corporate income tax rate on firm profitability, which is expected to negatively affect firm profitability. X_f,c,t_ is a vector including the two firm-level variables controlling for firm-size, LN (Assets) and firm-age, LN (Age).

The variable θ_t_ captures year-fixed effects to control for unobserved time-specific shocks that may affect all firms alike. We include also unobserved firm effects that is fixed, φ_f_, which allow to control for firm time-constant unobserved heterogeneity and focuses the analysis on the within-firm-country variation in profitability over time. Given that firm observations are per country, and thus firm-fixed effects are country-specific, then firm-fixed effects count for differences in consumer preferences for certain cars or brands in a given country.^32^ This explains why we avoid the inclusion of country-fixed effects as well, since it would produce the ‘collinearity with fixed effects’ problem. Country is fixed over time for the same firm, so the country indicators would be collinear with firm indicators.^33^ Therefore, we concluded to include only firm-fixed effects and time-fixed effects.

We rely on the traditional view of the fixed effects approach and assume that the unobserved effect is a parameter to be estimated for each firm, thus entering a dummy variable for each firm (cross-section observation) (Wooldridge, 2015). Similarly, we enter dummy variables for each year of our time-dimension and estimate a least square dummy variable model.

Since we match firm data with country level variables, then for firms in a country and year, all the variables that are set at the country level are the same. This may lead to more closely correlated error terms within this cell. Thus, two arguments speak against the basic OLS assumption that all errors are uncorrelated: (i) the fact that we observe firm year after year, so that observations of one firm are more correlated than across firms; (ii) two firms operating in the same year and in the same country may have more closely correlated errors than two firms in different countries. Taking into account this potential concern, in order to account for heteroscedasticity, which becomes an issue mostly in unbalanced panels (Besley and Rosen, 1999), and potential time-series dependence in the residuals, we report all estimates with robust standard errors, clustered at both firm and country level.^34^

### Results

Table 1 reports results of estimation of the empirical regression (5). In column 4, all the main variables of interest are included. In column 5 and 6 respectively, VAT and corporate income tax (CIT) is added to the regression, while in column 7, we include the two firm-level control variables, i.e., *LN(Assets)* and *LN(Age)*. The Motor Vehicle Registration Tax,—in thousands of Euros—enters in level form in all of the specifications. All specifications include firm and year-fixed effects. Standard errors are clustered at both firm and country level.

**Table 1:** Estimating the impact of Motor Vehicle Registration Tax (MVRT) on motor vehicle foreign-owned subsidiaries’ net profit margin (Lerner Index)

Dep. variable: lerner index [operating profit (EBIT)/operating revenue (turnover)]								
Model: OLS–FE								
Regressors	(1)	(2)	(3)	(4)	(5)	(6)	(7)	(8)
Herfindahl–Hirschman Index (HHI)	0.00102		0.00151**	0.00121*	0.000285	0.000355	0.000391	0.000557
	(0.000630)		(0.000648)	(0.000626)	(0.000720)	(0.000722)	(0.000722)	(0.000714)
Motor Vehicle Registration Tax (MVRT)		− 0.702***	− 0.646**	− 0.730***	− 11.44***	− 11.04***	− 10.89***	− 10.48***
		(0.251)	(0.261)	(0.253)	(2.645)	(2.811)	(2.800)	(2.797)
Motor Vehicle Registration Tax (MVRT) * HHI			0.00119		− 0.0203**	− 0.0200**	− 0.0199**	− 0.0186**
			(0.000918)		(0.00800)	(0.00808)	(0.00808)	(0.00802)
Specific MVRT *(dummy)*				2.049***	3.457***	3.406***	3.473***	2.350***
				(0.119)	(0.334)	(0.353)	(0.385)	(0.622)
Motor Vehicle Registration Tax (MVRT) * Specific MVRT					10.92***	10.47***	10.33***	10.01***
					(2.651)	(2.835)	(2.824)	(2.817)
Motor Vehicle Registration Tax (MVRT) * Specific MVRT * HHI					0.0209***	0.0207**	0.0206**	0.0193**
					(0.00796)	(0.00803)	(0.00803)	(0.00797)
Value added tax (VAT)						0.0366	0.0319	0.0633
						(0.0830)	(0.0837)	(0.0839)
Corporate income tax (CIT)							− 0.0203	− 0.0202
							(0.0281)	(0.0275)
LN (assets)								1.993*
								(1.021)
[LN(assets)]^2^								− 0.0935*
								(0.0482)
LN (age)								1.161*
								(0.592)
*Firm-fixed effects*	✓	✓	✓	✓	✓	✓	✓	✓
*Year-fixed effects*	✓	✓	✓	✓	✓	✓	✓	✓
*Clustering at firm and country level*	Yes	Yes	Yes	Yes	Yes	Yes	Yes	Yes
Observations	4896	4896	4896	4896	4896	4896	4896	4896
R-squared	0.636	0.638	0.638	0.638	0.644	0.644	0.644	0.646

The dependent variable in all the specifications is the Lerner Index, calculated as the ratio between operating profits net of depreciation (EBIT) and operating revenue (Turnover). Levels of significance are reported as ***p < 0.01, ** *p *< 0.05, * *p *< 0.1. *p* values are based on robust standard errors, double—clustered, i.e., at the firm-country level. Firms are observed during 2011–2019 (unbalanced sample). The unit of observation is firm—country—year. All estimations include firm-dummies and year-dummies effects. The Motor Vehicle Registration Tax enters in all the specifications in units of Euro, calculated as Motor Vehicle Registration Tax revenues, scaled by number of new motor vehicle registrations. For the construction of the interaction terms, the *Centering Variable approach* is used. Column (4) includes the full set of variables of interests, while in column (5) firm-level control variables, i.e., firm—size and firm-age, are added

We start with the interpretation of the results reported in column 3. First, in line with our expectations as well as with previous empirical papers’ findings, market concentration leads to more profitability. Thus, as the concentration level in the Sale of Motor Vehicles industry increases, this causes firms to exercise market power, becoming more efficient and generating larger profits. Evaluated at the sample mean of the Motor Vehicle Registration Tax, an increase in HHI by one unit increases the Lerner index by almost 0.002 percentage points, the effect being statistically significant at the 5% significance level.

Second, our estimation results suggest that the Motor Vehicle Registration Tax has a negative effect on motor vehicle multinationals’ profitability. Evaluated at the sample mean of the Herfindahl–Hirschman index, increasing the Motor Vehicle Registration Tax by one-unit, decreases the Lerner index by almost 0.65 percentage points, the effect being statistically significant at the 5% significance level. These numbers give us a first possibility to gauge the economic effects of MVRT.^35^ For a country like Spain, a doubling in the MVRT from currently around 400 Euros to 800 Euros would lead to a fall of the profit margin by roughly 0.25%. For Hungary, with a current MVRT of 800, doubling the MVRT would lead to a 0.5% fall in profit margins. This is not enormous, but certainly far from irrelevant. The coefficient in our interaction term, however, suggests that increases in the concentration level of the motor vehicle market seem not to influence the effect of Motor Vehicle Registration Taxes on firms’ profitability.

Important however, is the distinction we implement between countries where the Motor Vehicle Registration Tax is a *specific* tax and those where it is an *ad valorem* tax instead (see column 4), by including the *MVRT_specific(dummy)*. In column 5, we interacted this dummy with the Motor Vehicle Registration Tax, MVRT*MVRT_specific(dummy), as well as with the Herfindahl–Hirschman index (HHI), MVRT*MVRT_specific(dummy)*HHI. The coefficient on *Motor Vehicle Registration Tax (MVRT)*, i.e., [-11.44], indicates now the effect of the Motor Vehicle Registration Tax formulated as an ad valorem tax on the Lerner index. It suggests that, evaluated at the sample mean of the motor vehicle market concentration level, an increase in the ad valorem Motor Vehicle Registration Tax by one unit, decreases the Lerner index by 11.44 percentage point, the effect being strongly statistically significant. The *MVRT_specific(dummy)* enters the regression with a strongly statistically significant positive coefficient. The interaction term MVRT*HHI results negatively and statistically significant at the 5% significance level, suggesting that the negative effect of an increase of the ad valorem MVRT on firm profitability, is bigger in more concentrated markets. Hence, the effect of the MVRT on motor vehicle foreign-owned subsidiaries’ profitability depends on the degree of market concentration, in countries where the tax is formulated as an ad valorem tax. This would suggest that, as imperfect competition increases, the price-decrease effect of the ad valorem Motor Vehicle Registration Taxes increases, i.e., more of the tax burden is borne by firms through a motor vehicle price decrease, which reflects on a bigger negative effect of the MVRT on their profitability. It also points to different perceptions of particular formulation of a MVRT. A higher ad-valorem tax may move buyers away from more expensive motor vehicles with (typically) higher margins, which may not be the case for a specific tax.

Our results suggest a negative effect of the Motor Vehicle Registration Tax on the Lerner index in countries where it is formulated as a specific tax as well, although the size of the coefficient is much lower than in the ad valorem MVRT. Evaluated at the sample mean of the Herfindahl–Hirschman index, increases in the specific MVRT, decrease the profitability of motor vehicle foreign-owned subsidiaries by 0.53 percentage points [(-11.44) + 10.92], the effect being statistically significant at the 5% significance level. Differently from the ad valorem MVRT, the Herfindahl–Hirschman index does not affect the impact of the specific MVRT on our dependent variable. As the concentration level in the Sale of motor vehicles’ industry increases, the effect of the MVRT on firm profitability, in countries where it is formulated as a specific tax, does not change. This can be observed from the statistical insignificance of the linear combination of the coefficients on the respective interaction terms, MVRT*HHI and MVRT*MVRT Specific*HHI [(− 0.0203) + 0.0209]. While the statistical insignificance is an interesting observation from a policy perspective, as it suggests that unlike ad valorem Motor Vehicle Registration Taxes, specific Motor Vehicle Registration Taxes are neutral, i.e., their effect on firm profitability overcomes differences in the competition level in the motor vehicle industry across EU countries, on the other hand, the statistical insignificance may also be attributed to some extent to data availability, since in our dataset, there are only four countries where the MVRT is formulated as a specific tax, as compared to twelve of them where MVRT is an ad valorem tax.

Column 6 reports results of the empirical regression while controlling for the value added tax (VAT). The value added tax does not have an impact on motor vehicle MNEs subsidiaries’ Lerner Index. Potential explanation for the insignificant effect, might relate first to the fact that we have scarce variability in the value added tax within and between countries in our sample, and it changes only once for the majority of the rest of the countries. Second, policy-related reasons might lead to an insignificant effect of VAT on firm profitability, i.e., value added tax changes are usually anticipated several months in advance by governments and tax authorities’ announcements, giving somehow to the firms the possibility to already anticipate effects that upcoming changes might bring. Last, while VAT may be levied at the purchase of the new motor vehicle, it might then be declared in the country where the motor vehicle will be registered, leaving thus more room for international tax evasion of VAT on motor vehicles.

In column (7), we control for the effect of statutory corporate income tax on firm profitability as well. While the coefficient on the CIT is negative, it is statistically insignificant. Controlling for VAT and CIT does not change our main findings. The effect of the MVRT on motor vehicle foreign-owned subsidiaries’ profitability remains negative and statistically significant both for ad valorem MVRT and specific MVRT, with the coefficient being slightly lower for the ad valorem MVRT (10.89) and slightly higher for the specific MVRT [(− 10.89) + 10.33]. On the other hand, the effect of the Motor Vehicle Registration Tax on motor vehicle foreign-owned subsidiaries’ profitability depends on the degree of market concentration only in countries where the tax is formulated as an ad valorem tax (− 0.0199, 5% statistical significance).

In column (8) we add the two firm-level control variables, *LN (Assets)*_*c,t*_ and *LN (Age)*_*c,t*_. Both of them have plausible signs. As the motor vehicle foreign-owned subsidiaries become older, their profitability increases, with the effect of firm-age being only marginally statistically significant. The coefficient on *LN (Assets)* is positive and statistically significant at the 10% significance level. In line with Giroud and Mueller (2010), we experimented with squared terms for size, to capture possible non-linearities. The squared term for firm—size is negative and marginally significant, which implies that the relation between firm—size and Lerner Index is concave.

The rest of the results remain as in the previous columns. Evaluated at the sample mean of the Herfindahl–Hirschman index, the MVRT exerts a negative and strongly statistically significant effect on motor vehicle foreign-owned subsidiaries’ Lerner index, both when formulated as an ad valorem tax and as a specific tax. However, the negative effect is moderated by the concentration level in the Sale of motor vehicles’ industry in countries where the MVRT is formulated as an ad valorem tax. The negative effect of the ad valorem MVRT on motor vehicle foreign-owned subsidiaries’ profitability is higher in more concentrated markets.

Our findings suggest that first, increases in Motor Vehicle Registration Taxes across European Union countries negatively affect motor vehicle MNEs’ market performance in the Sale of Motor vehicles industry, both when they are formulated as an ad valorem tax on the net or gross price, and as a specific tax. The negative effect of these taxes on motor vehicles foreign-owned subsidiaries’ profitability may reflect a price-decrease effect. As registration taxes become higher, firms tend to offer lower pre-tax prices, in order to compensate for the higher tax effect. In addition, our results suggest that the role of imperfect competition as a moderator on the effect of the MVRT on firms’ market performance, depends on the structure of the tax, i.e., whether it is formulated as an ad valorem tax or as a specific tax. We found that the negative effect of the ad valorem MVRT increases as the market becomes less competitive. Thus, increases in the concentration level, increase the tax burden borne by motor vehicle sellers, which is reflected in lower profitability levels. On the other hand, market concentration level does not result to impact the effect of the registration tax on firm profitability in countries where the tax is formulated as a specific tax.

As highlighted in our theoretical framework, imperfect competition makes it particularly difficult to credibly predict the pass-through rate. Theoretically, we showed that firm *i*’s profits depend on own price elasticity of demand, elasticity of the cost function and cost function, as well as on the tax *t* on firm i’s motor vehicles and on the average tax *T* on competitors’ motor vehicles. Thus, it was challenging to make informative theoretical predictions about the effect of the Motor Vehicle Registration Taxes on firm profitability under imperfect competition. Only empirically, our results showed the effect of Motor Vehicle Registration Taxes on firm profitability, which in line with the standard economic theory of tax shifting, and with prior empirical evidence on the tax incidence of ad valorem and specific taxes (i.e., Delipalla and O’ Donnell, 2001; Bonnet and Réquillart, 2013; Carbonnier, 2013), suggested that in imperfect competition, the equivalence of specific and ad valorem taxes on price changes and on changes on outcomes other than prices, leaks away.

## Robustness

This section aims to show that our findings in the baseline regression are robust to several robustness checks. Results of the robustness analysis are reported in Appendix B.

In Table 4, we restrict the sample to motor vehicle foreign-owned subsidiaries operating in sixteen European Union countries, excluding firms operating in Portugal. The mean of Motor Vehicle Registration Tax in Portugal is almost three times larger than the sample mean of Motor Vehicle Registration Tax. Therefore, we want to know whether Portugal drives our findings. As reported in Table 4, all the coefficients preserve their signs and their significance levels. Market concentration increases firm profitability. The Motor Vehicle Registration Tax has a negative effect on Lerner index, both when formulated as ad valorem, as well as a specific tax. As the Sale of Motor Vehicles’ industry becomes less competitive and market concentration increases, the negative effect of the Motor Vehicle Registration Tax in countries where it is an ad valorem tax becomes larger.

As w**a**s shown in Fig. 1, Belgium is the only country in our sample whose Herfindahl–Hirschman index in the Sale of motor vehicles’ industry indicates a moderate concentration, with an average value between 2011 and 2019, of about 1786. In order to see whether the high value of HH Index in Belgium relative to other countries,—with within countries’ HHI average value lower than 1500,—drives our results, we perform a robustness check excluding motor vehicle MNEs’ subsidiaries located in Belgium. As reported in Table 5, the exclusion of Belgium does not affect our main findings.

When the coverage of firms varies over time, as is the case with the Orbis database, since HH Index relies on the distribution of market shares in an industry, changes in the coverage of firms might lead to artificial changes in the resulting concentration index. Discrete indexes, which define the industry concentration based on absolute numbers of the top-n firms, mitigates such issue.^36^ Although differences between the concentration ratio and the cumulative index—HHI, exist, we expect them to yield reasonably comparable results (Bailey and Boyle, 1971). In Table 6, we report results of the regression where the market concentration level is measured through the concentration ratio of the top-4 firms based on sales,—namely CR4,—operating in the Sale of Motor Vehicles’ industry in country *c* in year *t.* In line with our expectation, measuring the concentration level in the Sale of Motor Vehicles’ industry across European Union countries either through discrete measures, i.e., CR4, or through cumulative index, i.e., Herfindahl–Hirschman index, yield to reasonably comparable results. Although the coefficients are lower than in the baseline results, the MVRT negatively affects the foreign-owned subsidiaries’ Lerner Index. The negative effect is moderated by the concentration level in the Sale of motor vehicles’ industry in countries where the Motor Vehicle Registration Tax is formulated as an ad valorem tax, although the coefficient on the interaction term MVRT*HHI becomes now marginally significant.

In Table 7, we use another common measure as profitability proxy for firm market performance, i.e., return on assets (ROA). We measure ROA as the ratio between earnings before interest, taxes, depreciation and amortization and total assets. In line with Grullon et al., (2019), Herfindahl–Hirschman index, same as on firm Lerner index, exerts a negative effect on ROA. In line with our baseline results on Lerner index, we find an overall negative impact of Motor Vehicle Registration Tax on foreign-owned subsidiaries’ return on assets, while the negative effect of the ad valorem MVRT on ROA is larger as the motor vehicle industry becomes less competitive.

Finally, we conduct a robustness test calculating the Lerner index as *EBIT* divided by firm *sales*, instead of *EBIT* divided by *turnover*. None of our main findings change, while the effect of firm size on firm profitability becomes stronger.

## Conclusions

This study has analyzed the impact of taxes related to the purchase and registration of motor vehicles, i.e., Motor Vehicle Registration Tax (MVRT) and market concentration level on the market performance of multinational enterprises operating in the Sale of Motor Vehicles’ industry across seventeen European Union member states. We investigated empirically three questions: (i) what is the effect of the concentration level in the Sale of Motor Vehicles’ industry on the profitability of motor vehicle multinational enterprises operating in the EU motor vehicle industry; (ii) do the one-off Motor Vehicle Registration Taxes (MVRT) charged across European Union countries on the first registration of motor vehicles have an impact on motor vehicle multinationals’ market performance, measured through profitability; (iii) is the impact of Motor Vehicle Registration Taxes on motor vehicle sellers’ profitability moderated by the concentration level in the national motor vehicle market where customers register their motor vehicle?

Using unconsolidated firm-level data on foreign-owned subsidiaries operating in the Sale of Motor Vehicles’ industry across seventeen European Union member states, we investigated our questions in a panel with fixed effects OLS regression analysis. In line with prior empirical studies, our results showed first that a less competitive EU motor vehicle industry affects positively the profitability of motor vehicle MNEs’ subsidiaries. Second, we found a strongly statistically significant negative effect of MVRT increase on the profitability of foreign-owned subsidiaries operating in the Sale of Motor Vehicles’ industry, which reflects a price-decrease effect. As registration taxes become higher, firms tend to offer lower motor vehicle pre-tax prices, in order to compensate for the higher tax effect. The effect of the tax increase on firm profitability is deteriorating both in countries where it is an ad valorem tax on net or gross motor vehicle price, and where it is a specific tax. Finally, our regression results suggested that the role of imperfect competition as a moderator on the effect of MVRT on firms’ market performance depends on the structure of the tax. We found that market concentration level in the EU motor vehicle industry plays a role on the effect of Motor Vehicle Registration Tax increases on firm profitability, only in countries where these taxes are an ad valorem tax. The negative effect of an increase in the MVRT in these countries becomes higher as the market becomes less competitive.

Our study modestly contributes to several gaps consulted both in the literature on indirect taxes’ incidence, as well as on the literature on motor vehicle taxation in general. Instead of focusing on motor vehicle prices, we looked at the tax incidence on firm profitability; we conducted a cross-country analysis and exploited both the difference in Motor Vehicle Registration Tax’ rates and structure across EU countries, as well as differences in national motor vehicle markets’ concentration levels.

This paper addresses a relevant message to policymakers in EU. It adds to the current debates in Europe about the effects of the harmonization, which hinge crucially on the way in which prices relate to taxes. While our findings raise concerns over the integration of the European Union motor vehicle market, they advance a proposal to these concerns, suggesting that the structure of Motor Vehicle Registration Taxes may mitigate the negative effects of the incomplete motor vehicle market integration and the lack of harmonization of motor vehicle taxes across EU countries. In particular, when formulated as specific tax, Motor Vehicle Registration Taxes are neutral to market concentration differences across the member states. Unlike ad valorem Motor Vehicle Registration Taxes, specific taxes on motor vehicles avoid the amplifying of their negative effect on firms’ profitability due to less competition in the motor vehicle industry. Accordingly, specific Motor Vehicle Registration Taxes would be recommended from a policy perspective.

## Appendix

### Appendix A: Summary statistics and data visualization

See Tables 2 and 3.

**Table 2:** Summary statistics of the constructed variables and their underlying variables

Variables	Obs	Mean	Std. dev	Min	Max
Lerner Index	4896	.0128152	.0272031	− .0972082	.1815564
Herfindahl–Hirschman Index	4896	469.2413	534.9121	120.7752	1884.145
Concentration ratio (CR4)	4896	30.0179	11.49286	15.88257	56.89354
Motor Vehicle Registration Tax	4896	660.6904	550.2689	0	3364.714
LN (assets)	4896	10.29688	1.441262	6.570164	14.57817
Age	4896	29.16667	17.83493	2	128
LN (age)	4896	3.19692	.6071435	.6931472	4.85203
Leverage	4896	.6816574	.2237892	0	2.931052
Value added tax (VAT)	4896	20.9404	1.73253	18	27
Corporate income tax (CIT)	4896	26.47712	6.75939	9	34
*Underlying variables*					
Motor Vehicle Registration Tax revenues	4404	8.56e + 08	8.74e + 08	3,000,000	2.33e + 09
Nr. of new motor vehicle registrations	4795	1,261,452	1,142,419	18,137	4,017,059
Operating profits (EBIT)	4896	3401.468	10,638.36	− 91,335.9	140,354
Operating revenue (Turnover)	4896	291,137.1	698,944.7	812.549	1.08e + 07
Long term debt	4896	4425.278	24,934.15	0	558,158.4
Current liabilities	4896	58,826.07	148,747.4	0	2,011,963
Total assets	4896	96,631.09	217,111.7	713.487	2,143,979
EBITDA	4440	5161.374	15,768.55	− 80,485.66	242,500

**Table 3:** Variables’ construction and data sources

Variable	Construction	Source
Lerner index	Operating profit (EBIT)/operating revenue (Turnover)	Bureau van Dijk’s Orbis database
Motor Vehicle Registration Tax (MVRT)	Motor Vehicle Registration Tax revenues^a^/Nr. of new MV registrations^b^	^a^Eurostat Main National Accounts Tax Aggregates database; ^b^ACEA Pocket Guides 2011–2019
Firm age	Current year—Year of incorporation (item AGE_COMPANY)	Bureau van Dijk’s Orbis database
Leverage	(Long term debt + Current liabilities)/Total assets	
Herfindahl–Hirschman Index	*HHI*_c,t =_ ${\sum }_{i\epsilon s}^{Nt}(MS$_f,c,t_)^2^	
Concentration ratio (CR4)	CR4_c,t_ = ${\sum }_{f\epsilon s}^{4}MS$_*f,c,t.*_	
Firm market share (MS_f,c,t_)	Firm Sales/Total firms’ sales in the Sale of MV industry in country *c* in year* t*	
LN (assets)	LN (Total assets)	
Value added tax (VAT)		OECD Tax Database; OECD (2020), Consumption Tax Trends 2020: VAT/GST and Excise Rates, Trends and Policy Issues, OECD Publishing, Paris; ACEA Tax Guides 2011–2019

### Appendix B: Robustness checks

See Tables 4, 5, 6, 7, and 8.

**Table 4:** Sample restricted to sixteen countries, i.e., excluding Portugal

Dep. variable: lerner index							
Model: OLS–FE							
Regressors	(1)	(2)	(3)	(4)	(5)	(6)	(7)
Herfindahl–Hirschman Index (HHI)	0.00116*	0.00128**	0.00116*	0.000111	0.000132	0.000146	0.000311
	(0.000627)	(0.000632)	(0.000627)	(0.000684)	(0.000683)	(0.000682)	(0.000677)
Motor Vehicle Registration Tax (MVRT)	− 0.606**	− 0.562**	− 0.606**	− 15.35***	− 15.22***	− 15.16***	− 14.57***
	(0.253)	(0.261)	(0.253)	(3.744)	(3.904)	(3.895)	(3.891)
MVRT * HHI		0.000626		− 0.0358***	− 0.0357***	− 0.0357***	− 0.0335**
		(0.000950)		(0.0131)	(0.0132)	(0.0132)	(0.0131)
Specific MVRT *(dummy)*			1.991***	2.776***	2.770***	2.797***	1.673***
			(0.120)	(0.235)	(0.239)	(0.289)	(0.583)
MVRT * Specific MVRT				14.92***	14.78***	14.73***	14.23***
				(3.751)	(3.928)	(3.919)	(3.911)
MVRT * Specific MVRT * HHI				0.0358***	0.0357***	0.0357***	0.0335**
				(0.0131)	(0.0132)	(0.0132)	(0.0131)
Value added tax (VAT)					0.0115	0.0103	0.0426
					(0.0834)	(0.0841)	(0.0844)
Corporate income tax (CIT)						− 0.0068	− 0.00591
						(0.0288)	(0.0281)
LN (assets)							2.210**
							(1.067)
[LN (assets)]^2^							− 0.104**
							(0.0503)
LN (age)							1.163*
							(0.600)
Firm-fixed effects	✓	✓	✓	✓	✓	✓	✓
Year-fixed effects	✓	✓	✓	✓	✓	✓	✓
Clustering at firm and country level	Yes	Yes	Yes	Yes	Yes	Yes	Yes
Observations	4721	4721	4721	4721	4721	4721	4721
R-squared	0.629	0.629	0.629	0.636	0.636	0.636	0.639

Robust standard errors in parentheses *** *p *< 0.01, ** *p *< 0.05, * *p *< 0.1

**Table 5:** Sample restricted to sixteen countries, i.e., excluding Belgium

Dep. variable: lerner index							
Model: OLS—FE							
Regressors	(1)	(2)	(3)	(4)	(5)	(6)	(7)
Herfindahl–Hirschman Index (HHI)	0.00175**	0.00252***	0.00175**	0.000403	0.000458	0.000492	0.000656
	(0.000783)	(0.000941)	(0.000783)	(0.00123)	(0.00125)	(0.00125)	(0.00123)
Motor Vehicle Registration Tax (MVRT)	− 0.745***	− 0.992***	− 0.745***	− 6.298***	− 6.102***	− 5.937***	− 5.784***
	(0.257)	(0.308)	(0.257)	(1.696)	(1.814)	(1.793)	(1.800)
MVRT * HHI		0.00215		− 0.0208**	− 0.0206**	− 0.0206**	− 0.0191**
		(0.00160)		(0.00941)	(0.00954)	(0.00954)	(0.00941)
Specific MVRT *(dummy)*			2.059***	3.210***	3.180***	3.253***	1.999***
			(0.122)	(0.413)	(0.422)	(0.450)	(0.646)
MVRT * Specific MVRT				5.672***	5.441***	5.298***	5.233***
				(1.764)	(1.900)	(1.884)	(1.892)
MVRT * Specific MVRT				0.0210**	0.0208**	0.0208**	0.0191**
				(0.00867)	(0.00879)	(0.00878)	(0.00868)
Value added tax (VAT)					0.0218	0.0171	0.0561
					(0.0837)	(0.0844)	(0.0847)
Corporate income tax (CIT)						− 0.0231	− 0.0287
						(0.0281)	(0.0274)
LN(assets)							1.794*
							(1.083)
[LN(assets)]^2^							− 0.0869*
							(0.0518)
LN(age)							1.370**
							(0.624)
*Firm-fixed effects*	✓	✓	✓	✓	✓	✓	✓
*Year-fixed effects*	✓	✓	✓	✓	✓	✓	✓
*Clustering at firm and country level*	Yes	Yes	Yes	Yes	Yes	Yes	Yes
Observations	4230	4230	4230	4230	4230	4230	4230
R-squared	0.645	0.645	0.645	0.650	0.650	0.650	0.653

Robust standard errors in parentheses *** *p *< 0.01, ** *p *< 0.05, * *p *< 0.1

**Table 6:** Using the concentration ratio of the Top—4 firms based on *Sales*, as a proxy for the concentration level in the Sale of Motor Vehicles’ industry

Dep. variable: lerner index									
Model: OLS–FE									
Regressors	(1)	(2)	(3)	(4)	(5)	(6)	(7)	(8)	(9)
Concentration ratio (CR4)	0.0105		0.0153	0.0153 (0.0155)	0.0223	0.00972	0.0133	0.0135	0.0184
	(0.0156)		(0.0155)	(0.0155)	(0.0167)	(0.0187)	(0.0191)	(0.0191)	(0.0188)
Motor Vehicle Registration Tax (MVRT)		− 0.702***	− 0.718***	− 0.718***	− 0.785***	− 8.090***	− 7.622***	− 7.498***	− 7.283***
		(0.251)	(0.253)	(0.253)	(0.258)	(1.733)	(1.900)	(1.889)	(1.889)
MVRT * CR4					0.0241	− 0.351**	− 0.339*	− 0.336*	− 0.308*
					(0.0247)	(0.173)	(0.176)	(0.176)	(0.175)
Specific MVRT (dummy)				2.042***		3.452***	3.391***	3.442***	2.327***
				(0.120)		(0.347)	(0.361)	(0.391)	(0.628)
MVRT * Specific MVRT						7.491***	6.948***	6.840***	6.713***
						(1.760)	(1.948)	(1.938)	(1.932)
MVRT * Specific MVRT * HHI						0.361**	0.350**	0.348**	0.317*
						(0.169)	(0.172)	(0.172)	(0.171)
Value added tax (VAT)							0.0494	0.0456	0.0815
							(0.0847)	(0.0854)	(0.0858)
Corporate income tax (CIT)								− 0.0157	− 0.0156
								(0.0281)	(0.0274)
LN(assets)									2.106**
									(1.018)
[LN(assets)]^2^									− 0.0993**
									(0.0481)
LN(age)									1.156*
									(0.596)
*Firm-fixed effects*	✓	✓	✓	✓	✓	✓	✓	✓	✓
*Year-fixed effects*	✓	✓	✓	✓	✓	✓	✓	✓	✓
*Clustering at firm and country level*	Yes	Yes	Yes	Yes	Yes	Yes	Yes	Yes	Yes
Observations	4896	4896	4896	4896	4896	4896	4896	4896	4896
R-squared	0.636	0.638	0.638	0.638	0.638	0.642	0.643	0.643	0.645

Robust standard errors in parentheses *** *p *< 0.01, ** *p *< 0.05, * *p *< 0.1

**Table 7:** Using the return on assets (ROA) as profitability measure

Dep. variable: return on assets (ROA) [EBITDA/total assets]									
Model: OLS–FE									
Regressors	(1)	(2)	(3)	(4)	(5)	(6)	(7)	(8)	(9)
Herfindahl–Hirschman Index (HHI)	0.00212		0.00241	0.00241	0.00295*	8.81e−06	4.55e−05	4.96e−05	0.000700
	(0.00169)		(0.00168)	(0.00168)	(0.00167)	(0.00196)	(0.00199)	(0.00199)	(0.00200)
Motor Vehicle Registration Tax (MVRT)		− 2.125**	− 2.162**	− 2.162**	− 1.970**	− 26.88***	− 26.61***	− 26.52***	− 24.72***
		(0.940)	(0.941)	(0.941)	(0.980)	(7.177)	(8.004)	(7.986)	(7.996)
MVRT * HHI					0.00187	− 0.0431**	− 0.0430**	− 0.0430**	− 0.0389*
					(0.00249)	(0.0197)	(0.0199)	(0.0199)	(0.0199)
Specific MVRT (dummy)				4.890***		8.389***	8.346***	8.386***	6.052***
				(0.443)		(0.912)	(1.035)	(1.107)	(1.833)
MVRT * Specific MVRT						25.27***	24.95***	24.86***	23.02***
						(7.221)	(8.220)	(8.200)	(8.195)
MVRT * Specific MVRT * HHI						0.0436**	0.0435**	0.0436**	0.0396**
						(0.0196)	(0.0198)	(0.0198)	(0.0198)
Value added tax (VAT)							0.0261	0.0240	0.136
							(0.271)	(0.272)	(0.271)
Corporate income tax (CIT)								− 0.0133	− 0.0466
								(0.0795)	(0.0756)
LN (assets)									2.012
									(3.068)
[LN(assets)]^2^									− 0.171
									(0.143)
LN(age)									4.047**
									(1.643)
*Firm-fixed effects*	✓	✓	✓	✓	✓	✓	✓	✓	✓
*Year-fixed effects*	✓	✓	✓	✓	✓	✓	✓	✓	✓
*Clustering at firm and country level*	Yes	Yes	Yes	Yes	Yes	Yes	Yes	Yes	Yes
Observations	4437	4437	4437	4437	4437	4437	4437	4437	4437
R-squared	0.679	0.681	0.681	0.681	0.682	0.686	0.686	0.686	0.690

Robust standard errors in parentheses *** *p *< 0.01, ** *p *< 0.05, * *p *< 0.1

**Table 8:** Lerner index calculated as Operating profits (EBIT)/Sales

Dep. variable: lerner index [operating profit (EBIT)/sales]									
Model: OLS–FE									
Regressors	(1)	(2)	(3)	(4)	(5)	(6)	(7)	(8)	(9)
Herfindahl–Hirschman Index (HHI)	0.000979		0.00117*	0.00117*	0.00149**	0.000107	0.000183	0.000226	0.000436
	(0.000683)		(0.000679)	(0.000679)	(0.000691)	(0.000764)	(0.000768)	(0.000767)	(0.000763)
Motor Vehicle Registration Tax (MVRT)		− 0.698***	− 0.725***	− 0.725***	− 0.638**	− 11.07***	− 10.63***	− 10.45***	− 9.946***
		(0.259)	(0.261)	(0.261)	(0.268)	(2.853)	(3.010)	(2.996)	(3.007)
MVRT * HHI					0.00128	− 0.0226***	− 0.0223***	− 0.0222***	− 0.0205**
					(0.000930)	(0.00831)	(0.00840)	(0.00840)	(0.00833)
Specific MVRT (dummy)						3.256***	3.200***	3.281***	2.291***
						(0.418)	(0.431)	(0.460)	(0.666)
MVRT * Specific MVRT				2.081***		10.54***	10.05***	9.889***	9.461***
				(0.123)		(2.859)	(3.036)	(3.023)	(3.034)
MVRT * Specific MVRT * HHI						0.0235***	0.0232***	0.0232***	0.0214***
						(0.00829)	(0.00837)	(0.00837)	(0.00829)
Value added tax (VAT)							0.0398	0.0345	0.0699
							(0.0877)	(0.0886)	(0.0887)
Corporate income tax (CIT)								− 0.0243	− 0.0252
								(0.0298)	(0.0291)
LN(assets)									2.496**
									(1.100)
[LN(assets)]^2^									− 0.121**
									(0.0529)
LN(age)									1.058*
									(0.640)
*Firm-fixed effects*	✓	✓	✓	✓	✓	✓	✓	✓	✓
*Year-fixed effects*	✓	✓	✓	✓	✓	✓	✓	✓	✓
*Clustering at firm and country level*	Yes	Yes	Yes	Yes	Yes	Yes	Yes	Yes	Yes
Observations	4887	4887	4887	4887	4887	4887	4887	4,887	4,887
R-squared	0.631	0.633	0.633	0.633	0.633	0.637	0.637	0.638	0.640

Robust standard errors in parentheses *** *p *< 0.01, ** *p *< 0.05, * *p *< 0.1

### Appendix C

We extend the model by specifying the demand function D. Under the assumption of constant elasticity of substitution (CES) and constant marginal costs, we show that firm i’s profits depend on ε (own price elasticity of demand), η (elasticity of the cost function), c (cost function), tax *t* on firm i’s motor vehicles and, on the average tax *T* on competitors’ motor vehicles.

We start by defining Q = [$\sum_{i=1}^{n} {{q}_{i}}^{\frac{\varepsilon -1}{\varepsilon }}$] s.t. $\sum {p}_{i }{q}_{i}$ ≤ Y *(budget constraint)*

6 $$\frac{\mathrm{d Q}}{\mathrm{d }{\mathrm{q}}_{\mathrm{i}}}={\left[ \sum_{\mathrm{i}=1}^{\mathrm{n}} {{\mathrm{q}}_{\mathrm{i}}}^{\frac{\upvarepsilon -1}{\upvarepsilon }} \right] }^{1/\upvarepsilon -1}{{\mathrm{q}}_{\mathrm{i}}}^{-1/\upvarepsilon }-\Delta {p}_{i}=0$$

From (7), we aggregate until we derive the price index:

7 $$\begin{aligned} \left[ {{ }\mathop \sum \limits_{{{\text{i}} = 1}}^{{\text{n}}} {\text{ q}}_{{\text{i}}}^{{\frac{{{\upvarepsilon } - 1}}{{\upvarepsilon }}}} { }} \right]{ }^{ - 1} {\text{q}}_{{\text{i}}}^{{{\upvarepsilon } - 1/{\upvarepsilon }}} & = {\Delta }^{{1 - {\upvarepsilon }}} {\text{p}}_{{\text{i}}}^{{1 - {\upvarepsilon }}} \\ \left[ {{ }\mathop \sum \limits_{{{\text{i}} = 1}}^{{\text{n}}} {\text{ q}}_{{\text{i}}}^{{\frac{{{\upvarepsilon } - 1}}{{\upvarepsilon }}}} { }} \right]^{ - 1} \left[ {\mathop \sum \limits_{{{\text{i}} = 1}}^{{\text{n}}} {\text{q}}_{{\text{i}}}^{{\frac{{{\upvarepsilon } - 1}}{{\upvarepsilon }}}} } \right] & = {\Delta }^{{1 - {\upvarepsilon }}} { }\mathop \sum \limits_{{{\text{i}} = 1}}^{{\text{n}}} {\text{p}}_{{\text{i}}}^{{1 - {\upvarepsilon }}} \\ {\Delta }^{{{\upvarepsilon } - 1}} & = \mathop \sum \limits_{{{\text{i}} = 1}}^{{\text{n}}} {\text{p}}_{{\text{i}}}^{{1 - {\upvarepsilon }}} \\ {\Delta }^{ - 1} = \mathop \sum \limits_{{{\text{i}} = 1}}^{{\text{n}}} {\text{p}}_{{\text{i}}}^{{1 - {\upvarepsilon }\frac{1}{1} - {\upvarepsilon }}} & \equiv {\text{P}}\;\left( {price\;index} \right) \\ \end{aligned}$$

From ${\mathrm{Q}}^{1/\upvarepsilon }{{\mathrm{q}}_{\mathrm{i}}}^{-1/\upvarepsilon }=\frac{{\mathrm{p}}_{\mathrm{i}}}{\mathrm{P}}$, the demand function is:

8 $${\text{q}}_{{\text{i}}} = \left( {\frac{{{\text{q}}_{{\text{i}}} }}{{\text{P}}}^{{ - 1/{\upvarepsilon }}} } \right){\text{Q}}$$

In a monopolistic competition, the demand function changes w.r.t in a way such that FOC is satisfied:

9 $$\frac{d{q}_{i}}{d{p}_{i}}=-\upvarepsilon {{p}_{i}}^{-\varepsilon -1} {P}^{-\varepsilon }\mathrm{Q}$$

10 $$\mathrm{Therefore}, \frac{d{q}_{i}}{d{p}_{i}}\frac{{p}_{i}}{{q}_{i}}=\upvarepsilon$$

Assuming constant marginal costs, thus that $\frac{{dc}_{i}}{{dq}_{i}}$ = c for every firm *i*, then:

11 $$\begin{aligned} \mathop \sum \limits_{{{\text{i}} = 1}}^{{\text{n}}} {\text{p}}_{{\text{i}}}^{{1 - {\upvarepsilon }}} & = \left( {\frac{{\upvarepsilon }}{{{\upvarepsilon } - 1}}^{{1 - {\upvarepsilon }}} } \right){\text{c}}^{{1 - {\upvarepsilon }}} \mathop \sum \limits_{{{\text{i}} = 1}}^{{\text{n}}} \left( {1 + {\text{t}}} \right)^{{1 - {\upvarepsilon }}} \\ \left[ {\mathop \sum \limits_{{{\text{i}} = 1}}^{{\text{n}}} {\text{p}}_{{\text{i}}}^{{1 - {\upvarepsilon }}} { }} \right]^{{\frac{1}{1} - {\upvarepsilon }}} & = \frac{{\upvarepsilon }}{{{\upvarepsilon } - 1}}{\text{c}}\left[ {\mathop \sum \limits_{{{\text{i}} = 1}}^{{\text{n}}} \left( {1 + {\text{t}}} \right)^{{1 - {\upvarepsilon }}} } \right]^{{1/1 - {\upvarepsilon }}} \\ \end{aligned}$$

where $\sum_{\mathrm{i}=1}^{\mathrm{n}}{(1+\mathrm{t})}^{1-\upvarepsilon }] \equiv 1+\mathrm{T}$, is the tax index, where t is the tax on firm i’s motor vehicles and T is the average tax on competitors’ motor vehicles. As price index $\mathrm{P}=\frac{\mathrm{\varepsilon \eta }}{\upvarepsilon -1}(1-\mathrm{T})$, then

12 $$\frac{{\mathrm{p}}_{\mathrm{i}}}{\mathrm{P}}=\frac{(1+{\mathrm{t}}_{\mathrm{i})}}{(1+\mathrm{T})}.$$

Substituting in the demand function in (8),

13 $${\mathrm{q}}_{\mathrm{i}}={\left(1+{\mathrm{t}}_{\mathrm{i}}\right)}^{-\upvarepsilon }({1+\mathrm{T})}^{\upvarepsilon }\mathrm{Q}.$$

Therefore, firm i’s profits depend on ε (own price elasticity of demand), η (elasticity of the cost function), c (cost function), tax *t* on firm i’s motor vehicles and, on the average tax *T* on competitors’ motor vehicles:

14 $${\pi }_{i}=\frac{\mathrm{\varepsilon \eta }}{\upvarepsilon -1}{(1+{\mathrm{t}}_{\mathrm{i}})}^{-\upvarepsilon }{(1+\mathrm{T})}^{\upvarepsilon }\mathrm{c}$$

## Footnotes

1 All sources of market segmentations, such as the *type approval*, *national registration system* and *distribution system*, considered as explanations for price differentials in Europe, have more or less vanished (Dvir and Strasser, 2018). In 2011, the European Commission’s Directorate General for Competition stopped publishing the data set on car prices (EC data set), based on the conviction that, by 2011, major car price difference between Member States have disappeared. EC’s website offers the following reasoning for ending the survey: *“Between 1993 and 2011, the Commission has published annually the [...] Car Price Report. This report has been discontinued. When the report was launched, there were major car price differences among Member States, and it was much more difficult for consumers to compare prices across borders. Since then, the situation has improved greatly, in part due to enforcement action by the Commission, and also thanks to the increased availability of price information on the internet. This means there is no longer a need for the Commission to duplicate this information in the Car Price Report”* (European Commission, 2013)*.*

2 In the paper, the abbreviation ‘VAT’ states for Value Added Tax; the abbreviation ‘MVRT’ states for Motor Vehicle Registration Tax.

3 A motor vehicle sold in the EU is considered **new** if the customer takes possession of it less than six months from the date it first went on the road, or the vehicle has been driven less than 6,000 km. See https://europa.eu/youreurope/citizens/vehicles/motorvehicles/VAT-buying-selling-motorvehicles/index_en.html.

4 In the European Union they account for 30% of total tax revenue, or 12% of GDP (Benzarti and Carloni, 2019).

5 By and large, taxes are levied base on the following criteria: the price or value of the vehicle; the engine power or cylinder capacity; environmental impact, incl. polluting emissions, CO_2_ emissions and the type of fuel used; social considerations incl. preferential treatment of emergency vehicles, ambulances, vehicles for disabled people, vehicles for public transport; other specific criteria applying to commercial vehicles such as number of axles, cargo room, number of seats, etc.; weight; presence of safety equipment; air conditioning etc. It would be interesting to analyze whether a different structure of the tax have a differentiated impact on our results, but since almost every country adheres to a different system, this would be picked up by the country fixed effect. It is beyond the scope of this paper to speculate on possible implications.

6 E.g., brackets of fiscal horsepower, tax policy regarding diesel, etc.

7 VAT, sales taxes, and sector-specific indirect taxes.

8 Besley and Rosen (1999) is the first paper, which clearly highlighted the lack of the contribution of the empirical analysis on a better understanding of the theory of tax incidence: *“The lack of empirical evidence had left the government’s technical staff work on two typical assumptions: (1) tax shifting is the same for all goods and (2) that shifting is full, i.e., consumer bear the full burden. This has also been the assumption in most academic studies of sales tax incidence, where it was assumed that prices fully reflect taxes, so that the only important empirical question was how these prices increases affect member of different member groups” (Besley and Rosen, *1999*, p. 158).*

9 While in the same line, Sutton (1991) exceeds this, by showing that the presence of sunk costs such as advertising and R&D may result in declining industry profitability as concentration levels increase.

10 SCP (Structure-Conduct-Performance) studies typically measure market performance by using one of the three mentioned measures (Church and Ware, 2000, p.426).

11 *Economic Profits* are the difference between revenues and the opportunity cost of all inputs. In the long run, economic profits are an indicator of market power. A *Rate of Return Investment* is the ratio of earnings or income to investment. Rates of return used to measure profitability include the rate of return on assets and the rate of return on shareholder’s equity (investment). Given that the measure of the *Lerner Index* directly, as (P-MC/P) is not possible, since accounting data on MC is not usually available, the *price–cost margin* is used instead. It is defined as (P-AVC)/P, where AVC is average variable cost. *Tobin’s q* uses stock market valuations to assess economic profits. It is the ratio of the market value of the firm to the replacement cost of its assets (Church and Ware, 2000, pp. 426–428).

12 This approach is proposed in the work of De Loecker and Warzynski (2012). This methodology estimates mark-ups with a so-called “production function approach”, as no assumption is required on the shape of demand faced by companies and on how firms compete. In this framework mark-up is derived from the first order condition of the firm’s cost minimization problem with respect to the flexible input and corresponds to the ratio between the elasticity of output with respect to the flexible input, and the cost of the variable input as a share of the firm’s revenue (Calligaris et al*.,* 2018, p.8). See also Traina (2018) and De Loecker et al., (2020).

13 Based on the relationship between the scope of the undertakings of the businesses concerned, concentration can take various forms: horizontal concentration and non-horizontal (vertical and conglomerate) concentration. Because the evolution of horizontal concentration in the industry and its level, is a key factor characterizing the level of the competitive environment (European Commission, 2013), we focus on an analysis of horizontal concentration, where the undertakings concerned are actual or potential competitors in the same relevant market, thus producing the same or similar products and selling them under the same geographical conditions (Brezina et al., 2016). The result of horizontal concentration is a strengthening of the dominance of enterprises and a reduction in the number of firms operating in the given market.

14 United Nations. Statistical Division. (2008). International Standard Industrial Classification of all Economic Activities (ISIC) (No. 4). United Nations Publications.

15 Firms within more narrowly defined industries are more likely to compete with each other.

16 This class wholesale and retail sale of new and used vehicles: passenger cars including specialized passenger motor vehicles such as ambulances and minibuses, etc.; lorries, trailers and semi-trailers; camping vehicles such as caravans and motor homes. This class also includes wholesale and retail sale of off-road motor vehicles (jeeps, etc.). See United Nations. Statistical Division. (2008). International Standard Industrial Classification of all Economic Activities (ISIC) (No. 4). United Nations Publications, p. 180.

17 Two measures of seller concentration are commonly used in SCP (Structure-Conduct-Performance) studies, i.e., the Herfindahl–Hirschman index and concentration ratios (Church and Ware, 2000, p. 429).

18 HH Index is a convex function of market shares of all subjects in a relevant market (Brezina et al., 2016). It is simply the sum of the squares of market shares for all firms in the industry. Here, we calculate it by using unconsolidated accounts of active firms operating in the Sale of Motor Vehicles’ industry in a specific country and year*: **HHI*_c,t =_ ${\sum }_{i\epsilon s}^{Nt}(MS$_f,c,t_)^2^. HHI can result in two extreme values, a maximum value of one if the market supply is represented by a single operating entity, or a minimal value 1/n if all entities have equal market shares.

19 Given that the industry chosen includes both passenger vehicles and other heavy vehicles and motorcycles and given that passenger vehicles are usually subject to different tax schemes that heavy vehicles or motorcycles (although not necessarily), this can highlight a limitation of our analysis, since the concentration index might not reflect to the greatest extent the actual concentration in the passenger motor vehicle market.

20 When the coverage of firms varies across industries or over time, as is the case with the database that we use in the empirical analysis, since HH Index relies on the distribution of market shares in an industry, changes in the coverage of firms might lead to artificial changes in the resulting concentration index. Defining concentration based on absolute numbers of firms such as concentration ratio, of the top-n firms, mitigates such issue, since the data typically contain the largest firms in each industry throughout the periods, and the small number of groups included in the measures allows to manually check that important firms do not suddenly appear or disappear during the sample period as a result of coverage changes. For this reason, instead of HH Index, we use CR4 in our robustness checks. CR4 is measured as the share of the industry sales due to the four largest firms in Sale of Motor Vehicle’s industry in country *c* in year *t*: CR4 _c,t_ = ${\sum }_{f\epsilon s}^{4}MS$_*f,c,t..*_

21 In principle, either the *total sales* equal the sum of sales across all firms in the microdata at hand, or it can be obtained from an industry-level database, such as EU Klems or OECD Stan (Bajgar et al*.,* 2019). Given that the industry-level databases (e.g., OECD Stan and EU Klems) report at two-digit industry level, it would be trivial for our concentration measure, as long as the numerator and the denominator would not correspond to the same industry-level.

22 We checked the concentration trend in each country across the years, which seemed to manifest no indication for any conclusive overall increasing or decreasing trend in the concentration of the Sale of Motor Vehicles’ industry across countries.

23 The following definition of the ultimate owner is used: The ownership path from the firm to its ultimate owner is characterized by an ownership share of more than 50% and firms for which no shareholder is identified or for which ownership shares are unknown are also considered as ultimate owners.

24 As mark-ups are generally increasing with the firms’ size (see e.g., De Loecker and Eeckhout, 2017), this restriction on the sample should not affect the qualitative conclusions of the analysis (Calligaris et al*.,* 2018, p.10). However, we do not apply this restriction for the calculation of the concentration measures, since very small firms do not exert any impact on HHI values, and even less on concentration ratio.

25 The index of *Total new motor vehicle registration* includes passenger motor vehicles, light commercial vehicles up to 3.5t, commercial vehicles (trucks) over 3.5t, buses and coaches over 3.5t.

26 For Sweden and Germany, Eurostat Main National Accounts Tax Aggregates does not provide data on motor vehicle registration tax revenues. This, because Sweden charges neither a registration tax nor a registration fee on new motor vehicle registrations, while the average duty collected at the time of an initial registration in Germany amounts to only €26.30. Hence, we implement motor vehicle registration tax for these two countries manually.

27 Section 4.2., explained more in details the construction of the market concentration measure.

28 We consult this database with the European Automobile Manufacturers’ Association (ACEA) Tax Guides (2011–2019), in order to see whether all vehicles are subject to the standard VAT rate as reported in the OECD Tax database.

29 For the detection of extreme values: box plots, spikeplots (rootgrams) and histograms frequency distributions were used both for the constructed variables and their underlying variables.

30 We used leverage, studentized residuals, Cook’s D, and partial regression plots as a way to identify outliers as well as to find out whether they affect the fitted values of our dependent variable.

31 In order to prevent concerns for a correlation between the market concentration level and Motor Vehicle Registration Tax, we regressed the Herfindahl–Hirschman index on the Motor Vehicle Registration Tax, including year-fixed effects and country-fixed effects, and accounting for heteroscedasticity and serial correlation by clustering the standard errors at country level. We obtained a positive coefficient on the Motor Vehicle Registration Tax, which resulted statistically insignificant, neglecting a potential effect of the MVRT on market concentration level.

32 Accordingly, country-specific firm-fixed effects distinguish between for example Brand 1 in Country A and Brand 1 in Country B.

33 Furthermore, concentration is constant within a country, and the country-dummies would obviously outperform the concentration variable.

34 Presence of heteroscedasticity is suggested by scatterplots of fitted, predicted and residual values, Breusch-Pagan test, White test and a modified Wald statistic for group-wise heteroscedasticity in the residuals of a fixed-effect regression model. Presence of serial correlation (of different lags, up to T-1) is suggested by the Breusch-Godfrey test (which, unlike Durbin-Watson test, is less sensitive to the assumption of normality distribution of the residuals and allows us to test for serial correlation through a number of lags, hence besides just one lag).

35 Once we include interaction effects, the coefficient itself increases dramatically, but it is counteracted by interaction terms. We can think of these numbers presented here as rough averages.

36 This is possible since the data typically contain the largest firms in each industry throughout the periods, and the small number of groups included in the measures allows to manually check that important firms do not suddenly appear or disappear during the sample period as a result of coverage changes.
